# Impacts of Community-Based Natural Resource Management on Wealth, Food Security and Child Health in Tanzania

**DOI:** 10.1371/journal.pone.0133252

**Published:** 2015-07-17

**Authors:** Sharon Pailler, Robin Naidoo, Neil D. Burgess, Olivia E. Freeman, Brendan Fisher

**Affiliations:** 1 Clark University, Economics Department, Worcester, Massachusetts, United States of America; 2 World Wildlife Fund, Washington, District of Columbia, United States of America; 3 UNEP—World Conservation Monitoring Center, Cambridge, United Kingdom; 4 Centre for Macroecology, Evolution and Climate, The Natural History Museum, Copenhagen, Denmark; 5 ASB Partnership for the Tropical Forest Margins, World Agroforestry Centre, Nairobi, Kenya; 6 Gund Institute for Ecological Economics, Rubenstein School of Environment and Natural Resources, University of Vermont, Burlington, Vermont, United States of America; University of Florida, UNITED STATES

## Abstract

Community-based natural resource management (CBNRM) is a major global strategy for enhancing conservation outcomes while also seeking to improve rural livelihoods; however, little evidence of socioeconomic outcomes exists. We present a national-level analysis that empirically estimates socioeconomic impacts of CBNRM across Tanzania, while systematically controlling for potential sources of bias. Specifically, we apply a difference-in-differences model to national-scale, cross-sectional data to estimate the impact of three different CBNRM governance regimes on wealth, food security and child health, considering differential impacts of CBNRM on wealthy and poor populations. We also explore whether or not longer-standing CBNRM efforts provide more benefits than recently-established CBNRM areas. Our results show significant improvements in household food security in CBNRM areas compared with non-CBNRM areas, but household wealth and health outcomes in children are generally not significantly different. No one CBNRM governance regime demonstrates consistently different welfare outcomes than the others. Wealthy households benefit more from CBNRM than poor households and CBNRM benefits appear to increase with longer periods of implementation. Perhaps evidence of CBNRM benefits is limited because CBNRM hasn’t been around long enough to yield demonstrable outcomes. Nonetheless, achieving demonstrable benefits to rural populations will be crucial for CBNRM’s future success in Tanzania.

## Introduction

Community-based natural resource management (CBNRM) has been widely promoted as a strategy that aims to conserve biodiversity, while simultaneously enhancing rural livelihoods. The underlying theory argues that devolving control of natural resources to local communities improves households' access to and management of those resources, thereby improving the resource base and their benefits to communities [1,2]. Since the 1990s CBNRM has been implemented across the developing world [3,4] and continues to be an important and expanding conservation strategy [5]. Additionally, CBNRM provides a potential platform that other conservation strategies, such as Reducing Emissions from Deforestation and Forest Degradation (REDD+) and biodiversity offsets, can build from [5–9]. Despite the popularity of CBNRM there is limited evidence that it leads to improved conservation outcomes, and even less evidence of any socioeconomic benefits [5].

Estimating CBNRM impacts on human well-being is challenging, which perhaps explains the existing gap in the empirical literature. This challenge is primarily because conservation areas differ systematically in a number of ways from non-conservation areas. Locations for conservation activity [10–13] and communities involved in CBNRM are not randomly selected. There is evidence that different levels of resource governance differ systematically in their locations [14]. Communities that are already effectively managing natural resources may be more likely to be selected and funded for CBNRM initiatives [5] and areas set aside for conservation tend to be in areas with little competition for alternate land uses [11]. Therefore, areas chosen for CBNRM may have lower-value resources, or less productive potential [15], with higher-value areas allocated to other uses. Furthermore, conservation activities tend to geographically coincide with areas of high poverty; whether this is cause or effect has been explored by only a few, and this work generally focuses on protected areas, not CBNRM [10,12,13,16].

Because of the aforementioned systematic differences between CBNRM and non-CBNRM areas, there are challenges determining causal relationships between conservation activity and social and ecological outcomes. Traditional econometric approaches can be biased and yield inaccurate estimates since they do not control for systematic differences. Recently, scholars successfully applied matching approaches to determine social and ecological effects of protected areas [10,12–14,16]. Matching approaches identify “untreated” observations that have similar characteristics to “treated” observations, including characteristics that determine whether or not an observation receives treatment. In the conservation context, the “treatment” is the conservation activity, to be compared (matched) with similar “untreated” observations without any conservation activity. Matching facilitates an unbiased statistical test of the impact of conservation activity (treatment) compared to the situation that differs only in the lack of conservation activity.

While matching approaches are a useful tool for evaluating conservation impacts, matching has its limitations. Accurate, reliable matching estimates depend on the quality of the matches themselves, as well as the existence of comparable non-CBNRM areas. In the case of CBNRM impacts, it is difficult to know if all relevant factors are included in the estimation model. We often do not know what factors determine whether or not a community enters a CBNRM arrangement and therefore cannot match on those characteristics. Variables omitted from a matching model might represent important attributes that influence both CBNRM selection and food security or health outcomes; such an omission would result in biased estimates of CBNRM impact.

Here, we use a difference-in-differences estimation strategy to determine the impact of three different CBNRM approaches, Joint Forest Management (JFM), Community-based Forest Management (CBFM) and Wildlife Management Areas (WMAs), on wealth, food security and health outcomes in Tanzania. Difference-in-differences models estimate the impact of treatment by calculating the difference in change over time between a treated group and a non-treated group before and after treatment [17,18]. Since omitted variable or selection bias is present both pre and post-CBNRM, these biases do not influence estimates of the overall trends or differences over time between trends, assuming that unobserved variables do not manipulate the trends themselves. Without treatment, trends of treated and non-treated observations should be the same over time, thus any significant difference in trends is attributed to the treatment [18]. As such, we can determine: 1) if CBNRM in Tanzania has had an impact on participating communities' well-being and 2) to what extent the effect of CBNRM varies depending upon the type of CBNRM governance regime.

### CBNRM in Tanzania

Tanzania has been a major player in the CBNRM movement, providing an excellent opportunity to test if CBNRM activities impact the welfare of participating communities and how such impacts might differ across different types of CBNRM. In the late 1990's and early 2000's the Tanzanian government passed several pieces of legislation calling for devolution of natural resource governance to local populations through various CBNRM activities, CBFM, JFM and WMAs. Although community forest activities began as early as 1991, it was the 1998 Forest Policy and 2002 Forest Act that legally supported and facilitated community management and ownership of forests through JFM and CBFM [15,19]. Regulations for both CBFM and JFM were published in 2007; benefit-sharing arrangements were later published in 2014. The 1998 Wildlife Policy provided new opportunities for community management of wildlife resources [20]. WMA regulations were formulated in 2002 alongside the establishment of the first WMA, but a new Wildlife Policy in 2007 and ‘Non-Consumptive Utilization of Wildlife Regulations’ in 2008 recentralized many powers and benefits to the government [21]. Revised WMA regulations in 2012 promised to return more control and benefits to communities. There are now more than 105,000 km² of Tanzanian land currently under some form of CBNRM, managed or co-managed by over 2,400 villages [20,22].

Establishment and implementation of each CBNRM management regime differs, with varied levels of local community control and access to the natural resource base. CBFM areas are regimes where a village (or several villages) sets aside communal forests and develops management plans for government approval resulting in formal acknowledgment of their “ownership” of the land. JFM areas, in contrast, are typically established in forest areas formally reserved by the government. Although villages involved in JFM adopt management responsibilities for the land, having some input for forest management plans and improved access to resources, JFM land remains under government ownership. To designate a WMA, a village (or several villages) sets aside community-owned land for wildlife habitat, and develops management plans and regulations for the land; these plans are then approved by the government, giving managing communities formal “ownership” once the WMA is officially registered. According to law, CBFM communities have full authority over resource access and regulations, whereas JFM communities do not [20,23]. As previously mentioned, *de jure* community authority in WMAs has fluctuated over the past decade [21]. In practice, there is often considerable residual government control over all three types of reserves [20,21,24].

Different types of CBNRM typically vary in their location and administration. CBFM areas tend to occur in dry, miombo woodlands, while JFM areas tend to be designated in montane forest areas [15,23–25]. CBFM sites are small in size, and are generally managed by a single community (Table 1). Only a handful (~7%) of CBFM sites are managed by multiple villages while JFM sites, in most cases, (~85%) are managed by two or more communities. Therefore, JFM areas may also require some level of coordination among village and local governments. WMAs occur in areas important for wildlife habitat, such as near protected areas, wildlife corridors and buffer zones in savannah and miombo woodlands. WMAs are much larger than CBFM and JFM areas, and are always managed by multiple villages (Table 1), requiring coordination between multiple villages, local and central governments, and other actors such as private hunting companies [20]. Given differences in CBNRM governance regimes, structure and location characteristics, we expect to find variation in the level of benefits each CBNRM regime provides to communities.

**Table 1:** Descriptive statistics of JFM, CBFM and WMA areas.

	JFM	CBFM	WMA
Number of registered sites	37	381	17
Total area of registered sites (ha)	320,654	545,885	2,743,000
Total number of participating communities	169	406	148
Maximum area per site (ha)	134,511	39361	400,000
Minimum area per site (ha)	15	5	24,200
Median area per site (ha)	405	425	128,200
Maximum number of managing communities	18	9	24
Minimum number of managing communities	1	1	2
Median number of managing communities	4	1	7

Central to each of Tanzania's three CBNRM strategies is improving rural livelihoods, a primary objective in all of the different CBNRM types [2,15,20]. The inclusion of this objective is based upon the potential for CBNRM to improve rural livelihoods through a number of mechanisms. This includes providing financing for communal benefits such as pubic services and infrastructure [2,26,27] and positively contributing to households through use, harvest and sale of forest products [15,20,26]. Those involved in WMAs can additionally benefit through tourism opportunities, hunting revenue, and other income generating activities such as forestry and bee-keeping [20].

On the other hand, opportunity costs of CBNRM are high. Setting up and maintaining CBNRM areas requires time and financing, while monitoring and enforcing CBNRM translates to less time spent farming or on other income generating activities [15]. Furthermore, in the spirit of sustainable resource management, CBNRM can restrict community members' access to resources they are accustomed to accessing. Finally, crop raiding by wild animals can also be an issue, particularly in areas designed to protect wildlife habitat [20].

## Data & Methods

### Data

Data on wealth, household food security, and other household characteristics come from Tanzania's 2003, 2007 & 2011–12 HIV/AIDS and Malaria Indicator Survey [28–30]. Given CBNRM implementation is a lengthy process, and laws supporting CBNRM were passed in the late 1990’s and early 2000’s [15,20], we assume that 2003 is sufficiently early in the CBNRM process to act as a baseline for food security prior to CBNRM implementation. Data on health measures for children under five years old comes from Tanzania's 1999 & 2010 Demographic and Health Surveys (DHS) [31,32]. Likewise, we assume that 1999 is sufficiently early to serve as a measure of health outcomes pre-CBNRM implementation. Tanzania's HIV/AIDS and DHS surveys have the same sampling strategy; approximately 20 clusters (each cluster represents a village or collection of neighboring villages) are chosen per region and approximately 18 households are surveyed within each cluster. Since households in the capital city and offshore islands may be distinctly different from households in rural, mainland Tanzania (where CBNRM sites are located), we exclude clusters located in Dar es Salaam and the islands of Pemba and Unguja (collectively Zanzibar). We also exclude clusters that were not geolocated. Final sample sizes of the included surveys are listed in Table 2.

**Table 2:** Demographic Health Survey (DHS) sample sizes by year.

Survey year	Number of clusters	Number of observations
1999	129	2,111 children under 5
2003	319	6,016 households
2007	350	5,802 households
2010	348	5,736 children under 5
2011–12	467	7,825 households

**Fig 1:**
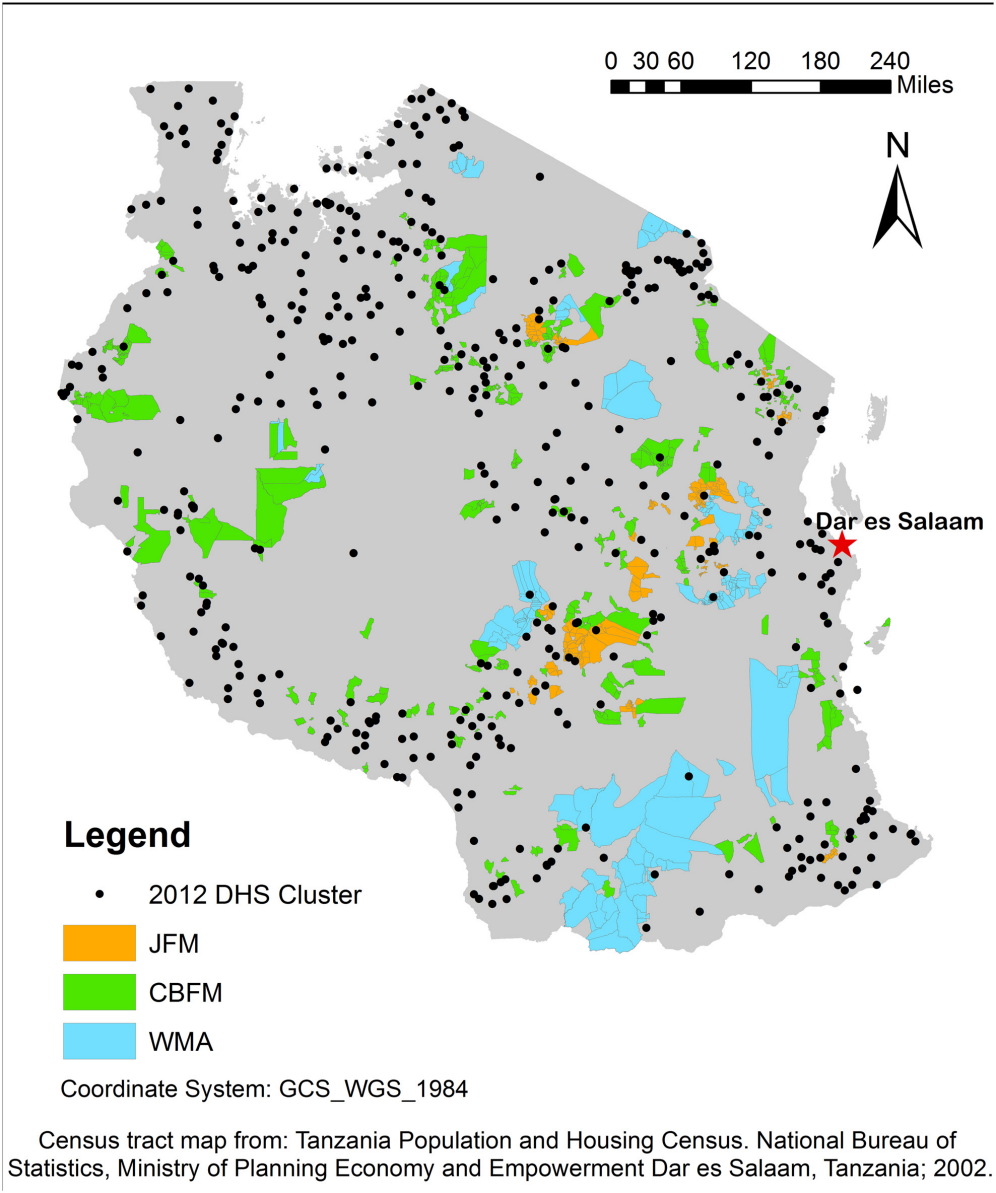
Census tracts of JFM, CBFM and WMA villages with 2012 DHS cluster GPS points. This map shows census tracts of villages participating in signed JFM and CBFM areas and registered WMAs. GPS points for 2012 DHS clusters indicate location of DHS survey sites relative to census tracts with CBNRM.

This study uses the most current, available lists of “signed” JFM and CBFM sites and registered WMAs derived from official sources [20,24,25]. Because official designation is a lengthy, bureaucratic, resource-intensive process, generally financed and facilitated by international organizations, there are many CBNRM areas that are “in process”, not yet officially signed or registered [15,20,22]. As of 2011, there were 381 signed CBFM areas totaling 545,885 ha managed by 406 villages and 38 JFM areas totaling 320,654 ha managed by 169 villages (Table 1). As of 2014 there were 17 registered WMAs, totaling 2,743,000 ha managed by 148 villages [20](Table 1). We matched village names from these lists to villages listed in Tanzania's 2002 national census shapefile, and were able to match 364 of 406 CBFM villages (90%), 160 of 169 JFM villages (95%), and 136 of 148 WMA villages (92%) to census tract polygons in the shapefile (Fig 1).

HIV/AIDs and DHS survey clusters are geolocated with GPS points that are up to 5 km away from the actual survey location to maintain respondent confidentiality. We therefore consider households that fall within 5 km of a CBNRM census tract to be within that CBNRM area. Our CBNRM sample could therefore include some non-CBNRM households. Since the 5 km displacement is randomly designated, the inclusion of non-CBNRM households would only bias against finding a significant difference in our estimates. For robustness, we test the base model using only households that directly intersect the census tract of villages involved in CBNRM (0 km distance). There are likely to be fewer non-CBNRM households included in this smaller CBNRM sample and should produce difference-in-differences estimates similar to those calculated using the sample with the 5 km buffer.

Data on slope, elevation and aridity come from the Consultative Group on International Agricultural Research [33]. Land use in 2002 is from the International Livestock Research Institute [34] and state-managed protected area locations and shapes are from the March 2014 version of the World Database on Protected Areas [35]. DHS data provides important information on village characteristics such as urban/rural status and distance from market. Tanzania's 2002 National Census [36] supplies data on population characteristics including population density, percent economically active and percent voting population.

### Dependent Variables

Our dependent variables include measures for food security, wealth and health outcomes.

The household wealth index is a standardized wealth score based on the distribution of wealth factor scores across surveyed households. Wealth factor scores are constructed based on household assets and construction materials, taking into account urban/rural location and access to public services and markets. Wealth factor scores are combined and adjusted based on specific characteristics and location to produce a “nationally applicable” wealth index metric that has mean zero and standard deviation of one (see S1 Text for a detailed description of wealth index) [30,37].

Food security indicators include household-level responses to the following survey questions [30]:

- *How many meals does your household usually have per day?*
- *In the past week*, *on how many days did your household eat meat or fish?*
- *How often in the last year did you have problems in satisfying the food needs of the household (never = 1*, *seldom = 2*, *sometimes = 3*, *often = 4*, *always = 5)?*

Health outcome data come from height and weight measurements for children under five years of age at the time of the survey. Height and weight data were used to calculate weight-for-age, height-for-age and weight-for-height using growth standards developed by the World Health Organization [32,38]. These indexes are often used as indicators of slowed growth and/or malnourishment. Weight-for-age or height-for-age z-scores that are two standard deviations below the mean are considered evidence of children being underweight or stunted (respectively). Weight-for-height z-scores more than two standard deviations below the mean are evidence of wasting and malnourishment [32].

### Difference-in-differences models

The difference-in-differences model we estimate is:

$$Y=\alpha +\beta_{1}CBNRM+\beta_{2}YrPostCBNRM+\beta_{3}CBNRM*YrPostCBNRM+\beta_{4}X+\varepsilon$$

Where Y is the dependent variable, in this case wealth, food security or health outcomes. CBNRM is a “treatment” dummy variable indicating whether or not an area is JFM, CBFM or WMA; this variable captures initial differences between CBNRM and non-CBNRM observations prior to policy implementation. YrPostCBNRM is a dummy variable for years post-CBNRM; this variable captures variations in Y across time that influence both CBNRM and non-CBNRM observations. For food security measures we include year dummies for 2007 and 2012, for health outcomes we include a year dummy for 2010. A set of covariates controlling for regional, household, community, geographic and biophysical characteristics is captured in X. The interaction term CBNRM*YrPostCBNRM is the difference-in-differences estimator that represents the difference between the pre-post dependent variable values of households involved in CBNRM and those not involved in CBNRM. The coefficient on this interaction term is equivalent to:

$$\beta_{3}=({\bar{Y}}_{CBNRMpost}-{\bar{Y}}_{CBNRMpre})-({\bar{Y}}_{nonCBNRMpost}-{\bar{Y}}_{nonCBNRMpre})$$

This coefficient is the difference in CBNRM areas before and after CBNRM implementation minus the difference in non-CBNRM areas before and after CBNRM implementation. The difference-in-differences estimator captures the difference in trends between households in JFM, CBFM and WMA areas versus non-CBNRM areas for each of the dependent variables. If there is no difference in trends between CBNRM areas and non-CBNRM areas then the coefficient *β*_3_ will not be significantly different than zero.

Model specifications for wealth, food security and health outcomes include characteristics that predict whether or not an area is CBNRM, as well as characteristics that influence the outcome of interest (Tables 3, 4 and 5). Therefore, a number of control variables are included to strengthen our model.

**Table 3:** Summary statistics of covariates included in food security and wealth models.

	No CBNRM	JFM	CBFM	WMA
Variable	obs = 16,330	obs = 872	obs = 2,388	obs = 734
Meals/day	2.035	1.895	2.046	2.062
Meat/fish/week	2.497	2.68	2.519	2.66
Problems meeting food needs	1.311	1.179	1.078	1.416
Wealth Index	-0.153	-0.204	-0.316	0.168
Number household members	5.311	4.74	5.372	4.692
Number children under 5	1.07	0.826	1.067	0.928
Max number years education	6.187	6.323	6.249	6.267
Single adult head of hh	0.147	0.188	0.149	0.202
Female head of hh	0.242	0.287	0.247	0.236
Regional avg 1999 wealth	-0.243	-0.086	-0.165	-0.247
Within 30km protected area	0.342	0.294	0.36	0.645
Within 5km forest reserve	0.333	0.852	0.467	0.439
Urban	0.186	0.213	0.094	0.324
Nearest market (km)	18.498	19.22	24.872	27.668
District-level population density	185.166	77.811	45.344	578.984
Percent economically active population	0.555	0.552	0.537	0.586
Percent voting population	0.493	0.49	0.478	0.524
Percent bushland	0.209	0.225	0.192	0.082
Percent cultivated land	0.324	0.37	0.293	0.379
Percent grassland	0.169	0.139	0.193	0.109
Percent woodland	0.204	0.133	0.268	0.243
Percent natural forest	0.02	0.121	0.041	0.015
Elevation (m)	1,052.556	1,198.208	1,132.263	969.62
Slope (% rise)	2.88	3.853	3.03	2.677
Aridity index	6,392.264	7,135.691	6,019.093	6,295.517

Summary statistics for all covariates for household level observations, pooled across years 2003, 2007 and 2012.

**Table 4:** Summary statistics for covariates included in the health model.

	No CBNRM	JFM	CBFM	WMA
Variable	obs = 6,444	obs = 314	obs = 846	obs = 308
Child age (months)	29.033	30.771	30.526	30.25
Female	0.501	0.541	0.515	0.513
Shared toilet	0.211	0.28	0.246	0.351
Tap water	0.189	0.264	0.273	0.373
Wealth Index	-0.305	-0.384	-0.405	-0.118
Number household members	7.541	6.742	6.468	7.315
Number children under 5	2.23	1.914	1.961	1.916
Max number years education	7.691	7.538	7.531	7.899
Single adult head of hh	0.057	0.076	0.069	0.023
Female head of hh	0.18	0.207	0.167	0.107
Regional avg 1999 wealth	-0.276	-0.072	-0.163	-0.225
Within 30km protected area	0.203	0.395	0.208	0.438
Within 5km forest reserve	0.349	0.952	0.498	0.458
Urban	0.156	0.124	0.104	0.214
District-level population density	160.522	95.412	48.922	494.306
Percent economically active population	0.541	0.543	0.532	0.569
Percent voting population	0.48	0.486	0.476	0.508
Percent bushland	0.218	0.262	0.214	0.138
Percent cultivated land	0.342	0.31	0.181	0.334
Percent grassland	0.157	0.086	0.171	0.081
Percent woodland	0.211	0.182	0.34	0.242
Percent natural forest	0.014	0.143	0.069	0.09
Elevation (m)	1,109.181	1,062.07	1,101.278	972.734
Slope (% rise)	2.719	3.328	3.132	2.441
Aridity index	6,209.609	6,872.019	6,422.725	6,272.99

Summary statistics for all covariates for individual-level observations (children less than five years of age), pooled across years 1999 and 2010.

**Table 5:** Summary statistics for health outcome dependent variables by year and CBNRM type.

	No CBNRM	JFM	CBFM	WMA
Dependent variables—health 1999	obs = 1,652	obs = 75	obs = 176	obs = 79
Height/age Z-score 1999	-1.779	-2.315	-2.193	-1.98
Weight/age Z-score 1999	-1.28	-1.784	-1.678	-1.49
Height/weight Z-score 1999	-0.236	-0.504	-0.432	-0.321
	No CBNRM	JFM	CBFM	WMA
Dependent variables—health 2010	obs = 4,792	obs = 239	obs = 670	obs = 229
Height/age Z-score 2010	-1.723	-1.921	-1.916	-1.488
Weight/age Z-score 2010	-0.958	-1.206	-1.145	-0.783
Height/weight Z-score 2010	0.039	-0.18	-0.081	0.07

Summary statistics for health outcome dependent variables are displayed separately for 1999 and 2010 since the WHO weight and height measurement system changed in 2006 [31,37].

For biophysical and spatial characteristics we include variables such as aridity, slope, and elevation, to capture agricultural capacity of the surrounding area and their influence on the location of conservation activities [10,11,14]. We also include the proportion of land in the surrounding census tract that is bushland, grassland, cultivated land, natural forest, and woodland forest, as land use can influence rural livelihoods as well as CBNRM location [26,39].

Geographic and community characteristics such as population density, distance to nearest market and a rural/urban dummy variable to capture accessibility of goods and services, are included. The DHS defines urban areas as large cities (capital cities and cities with over 1 million population), small cities (population over 50,000), and towns (other urban areas); rural areas are assumed to be countryside. Variables such as percent economically active and percent voting population per census tract are used to control for community characteristics that may influence participation in CBNRM. Proximity to traditional protected areas can also influence household wealth and food security [10,13]. Since CBNRM areas may tend to occur close to traditional protected areas, and because protected areas have been found to benefit communities up to 15–45 kilometers away [40,41], we include dummy variables for households within 30 kilometers of a protected area. Government-owned forest reserves also influence household access to forest resources, although at a smaller geographic scale than protected areas [42], therefore we include a control variable for sites within 5 km from government forest reserves. To control for any initial wealth differences in CBNRM location selection, the 1999 average regional wealth index is included along with regional fixed effects to control for regional differences.

Additionally, we control for household characteristics such as the number of household members and number of children under five years old. The number of household members may influence wealth depending up on the proportion of working individuals; likewise a large number of children under five means a large number of dependents, which may also influence household wealth. Female and single-adult heads of household, who may be disproportionately disadvantaged and have different food security outcomes, are also controlled for [43]. Since education levels influence earnings (and vice versa), we include the maximum number of years of education earned by an individual within a household to control for education. The food security and health models also control for wealth index, since household wealth impacts food security [43].

The health model includes additional controls for the child's sex and age in months as well as environmental health variables such as sharing a toilet with other households and tap water as the primary water source. Distance from nearest market is not observed in 1999 and is not included in the health model. Note that the weight and height indicators in the 2010 DHS use the new (2006) World Health Organization health standards [32,38]. These new measures are not necessarily comparable to 1999 weight and height measures. So long as the new (2006) system of measurement does not differentially impact CBNRM areas, then differences in health measures between 1999 and 2010 will be captured in the year dummy variable and not influence our difference-in-differences estimate.

To explore heterogeneity in food security and health effects of CBNRM, we adapt our model to a triple-differences model that examines the differential effect of CBNRM on observations with wealth indexes greater than or equal to the national average, versus those with less-than-average wealth indexes. Since there is evidence that the wealthy benefit more from CBNRM in Tanzania than the poor [44–46], we expect to find fewer CBNRM benefits among poor groups. We also modify the base model to include a variable that captures the effect of CBNRM areas that were initiated after the year 2005. Well-established CBNRM areas have been shown to be more effective at producing benefits to communities [47]. Given that it takes as many as 6–7 years to establish CBNRM sites in Tanzania [20,48], CBNRM areas initiated after 2005 may show fewer benefits in 2012 as those initiated before 2005.

## Results

### Base model

**Fig 2:**
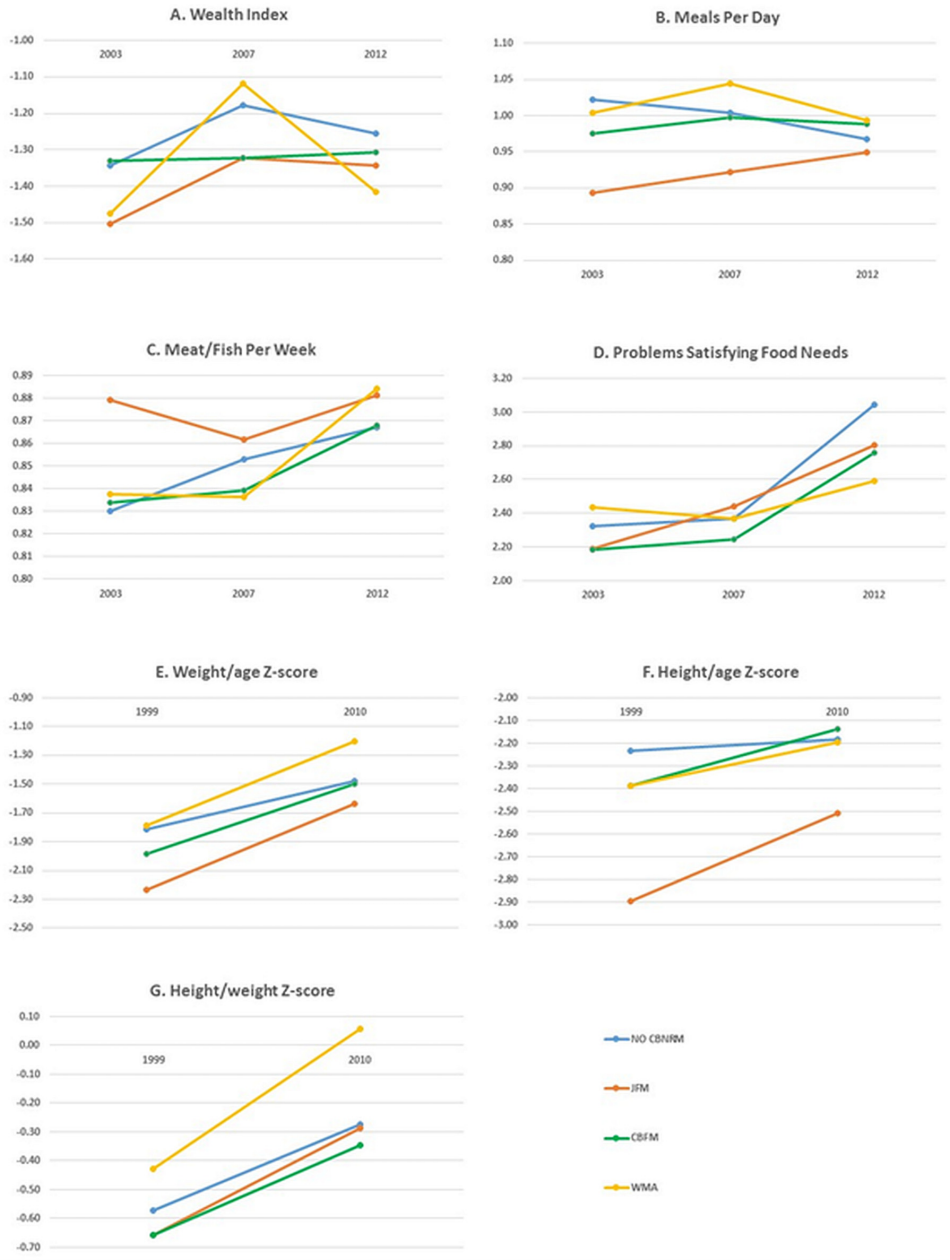
JFM, CBFM, WMA and non-CBNRM effects over time for each dependent variable, after controlling for household, community, geographic and biophysical characteristics. Difference-in-difference estimates for (A) wealth, (B) meals/day, (C) meat/fish/week, and (D) problems satisfying food needs are calculated for years 2003, 2007 and 2012. Estimates for (E) weight/age Z-scores, (F) height/age Z-scores and (G) height/weight Z-scores are calculated for years 1999 and 2010. The non-CBNRM coefficient in 2003 is the regression constant, in 2007 it is the constant plus the yr2007 dummy, in 2012 it is the constant plus the 2012 dummy. CBNRM types include the constant, the CBNRM dummy, the year dummies and the CBNRM*year interaction term.

Difference-in-differences coefficients are displayed in Table 6. Fig 2 and Tables 7 and 8 show coefficients for CBNRM and non-CBNRM types by year after controlling for household, community, geographic and biophysical characteristics, along with the difference in coefficients for wealth and food security measures between 2003 and 2012 (Table 7) and health outcomes between 1999 and 2010 (Table 8). Full model results are included in supporting information (S1–S7 Tables). Note that difference-in-differences models estimate differences in change over time between two groups, not the absolute differences within or between groups. Even if a variable is increasing over time, its difference-in-differences coefficient can be negative if it is increasing at a slower rate than the non-CBNRM comparison group.

**Table 6:** Differences-in-differences model results for wealth, food security and health outcomes.

	JFM	CBFM	WMA
Wealth index	0.0675	-0.0624**	-0.0278
	(0.0481)	(0.0294)	(0.0702)
Meals/day	0.109**	0.0659**	0.0403
	(0.0495)	(0.0292)	(0.0545)
Meat/fish per week (# of times)	-0.0358**	-0.00310	0.0108
	(0.0153)	(0.0107)	(0.0187)
Problems satisfying food needs last year	-0.101	-0.141**	-0.565***
	(0.0849)	(0.0584)	(0.106)
Weight/age	0.2544*	0.1508	0.2440
	(0.1518)	(0.1045)	(0.1671)
Height/age	0.3144*	0.1836	0.1329
	(16.89)	(12.32)	(0.1757)
Weight/height	0.08729	0.02883	0.1915
	(0.1648)	(0.1023)	(0.1631)

This table shows the difference-in-differences estimates for each of the CBNRM types on each dependent variable. The difference-in-difference estimate is the coefficient on the CBNRM*YrPostCBNRM variable, which captures the effect of CBNRM on the dependent variable in years post-CBNRM implementation. Robust standard errors are in parentheses.

*** indicates significance at the 1% level,

** at the 5% level, and

* at the 10% level.

**Table 7:** CBNRM and non-CBNRM wealth and food security effects by year with 2003–2012 differences calculated (1999–2012 differences for wealth index).

	2003	2007	2012	2003–2012 difference
Wealth index				
No CBNRM	-1.342	-1.179	-1.254	0.088
JFM	-1.502	-1.324	-1.344	0.158
CBFM	-1.330	-1.322	-1.306	0.024
WMA	-1.476	-1.118	-1.414	0.062
Meals/day				
No CBNRM	1.022	1.004	0.967	-0.055
JFM	0.893	0.922	0.949	0.056
CBFM	0.976	0.998	0.988	0.012
WMA	1.003	1.044	0.993	-0.010
Meat/week				
No CBNRM	0.830	0.853	0.867	0.037
JFM	0.879	0.862	0.881	0.002
CBFM	0.834	0.839	0.868	0.034
WMA	0.838	0.836	0.884	0.047
Frequency food problems				
No CBNRM	2.320	2.366	3.042	0.722
JFM	2.186	2.441	2.803	0.617
CBFM	2.184	2.241	2.756	0.572
WMA	2.435	2.367	2.588	0.153

The resulting estimates of JFM, CBFM, WMA and non-CBNRM households after controlling for household, community, geographic and biophysical characteristics. The non-CBNRM estimate for food security and health indicators in 2003 is the regression constant, in 2007 it is the constant plus the 2007 dummy coefficient, in 2012 it is the constant plus the 2012 dummy coefficient. CBNRM estimates include the constant, the CBNRM dummy, the year dummies and the CBNRM*year interaction term coefficients.

**Table 8:** CBNRM and non-CBNRM childhood health effects by year, with 1999–2010 differences calculated.

	1999	2010	1999–2010 difference
Weight/age Z-score			
NO CBNRM	-1.817	-1.478	0.340
JFM	-2.233	-1.633	0.600
CBFM	-1.988	-1.498	0.490
WMA	-1.785	-1.202	0.583
Height/age Z-score			
NO CBNRM	-2.235	-2.183	0.052
JFM	-2.897	-2.509	0.388
CBFM	-2.389	-2.139	0.251
WMA	-2.386	-2.196	0.190
Height-weight Z-score			
NO CBNRM	-0.571	-0.274	0.298
JFM	-0.659	-0.286	0.373
CBFM	-0.660	-0.346	0.313
WMA	-0.428	0.056	0.484

The resulting estimates of JFM, CBFM, WMA and non-CBNRM children under five years of age after controlling for household, community, geographic and biophysical characteristics. The non-CBNRM estimate in 2003 is the regression constant, in 2007 it is the constant plus the yr2007 dummy coefficient, in 2012 it is the constant plus the 2012 dummy coefficient. CBNRM estimates include the constant, the CBNRM dummy, the year dummies and the CBNRM*year interaction term coefficients.

After controlling for the effect of household, geographic and biophysical characteristics, the average wealth index in non-CBNRM households was higher by 0.09 in 2012 compared with 2003 (Tables 6 and 7, Fig 2a). In JFM areas, household wealth index increased 0.16 between 2003 and 2012 and was not significantly different than the change that occurred in non-CBNRM areas (Tables 6 and 7). Households in CBFM areas in 2003 have higher wealth indexes than households in JFM, WMA and non-CBNRM areas, suggesting CBFM tended to be established in relatively wealthier areas. In CBFM areas the average wealth index increased only 0.02; a trend that was significantly lower than non-CBNRM households. Despite a significant increase in average wealth index for WMA households between 2003 and 2007, the average wealth index in WMAs in 2012 was only 0.06 greater than in 2003; a trend not significantly different than non-CBNRM areas. Wealth model results for all CBNRM areas are robust when including wealth outcomes from the 1999 DHS survey.

Non-CBNRM households initially consumed more meals per day on average than households in CBNRM areas. The average number of meals per day consumed in non-CBNRM households decreases 0.06 meals per day between 2003 and 2012 (Tables 6 and 7, Fig 2b). JFM and CBFM households on average experience an increase in the number of meals consumed per day during the same time period, 0.06 meals per day and 0.01 meals per day respectively. These trends are significantly different than meal consumption in non-CBNRM areas. Despite a significant increase in meals per day consumed for WMA households in 2007 (0.04), the overall change between 2003 and 2012 indicates that the average number of meals per day has actually fallen 0.01 meals per day, which is not a significantly different trend than non-CBNRM areas.

In 2003, non-CBNRM households consumed meat less frequently than households in CBNRM areas with households in JFM areas consuming meat most frequently of all CBNRM types (Tables 6 and 7, Fig 2c). By 2012, households in non-CBNRM, CBFM and WMA areas consumed meat more frequently than in 2003 with meat consumption remaining the same in JFM households. Because meat consumption in JFM areas did not change between 2003 and 2012, yet increased in the non-CBNRM comparison group, the trend in meat consumption over time in JFM households is significantly negative.

CBNRM and non-CBNRM groups had similar average frequency in problems meeting food needs in 2003 after controlling for household, geographic and biophysical characteristics (Tables 6 and 7, Fig 2d). Although all groups experienced an increase in frequency in problems meeting food needs by 2012, non-CBNRM households experienced the largest increase (0.72). This increase was significantly smaller in CBFM (0.57) and WMA (0.15), but not in JFM (0.62).

Health outcomes demonstrate a positive trend for all groups between 1999 and 2010 (Tables 6 and 8, Fig 2e–2g). Across measured health outcomes for children, the only trends between 1999 and 2010 that are significantly different than non-CBNRM are height-age and weight-age ratios for children in JFM areas. Children in JFM areas have the lowest average values for height-age and weight-age ratios in 1999. In 2010, JFM children still have lower average values than other groups for height-age and weight-age ratios, but they seem to be “catching up” to the other groups with JFM children's height-age and weight-age ratios increasing significantly more than non-CBNRM children between 1999 and 2010. Trends in other health outcomes in other CBNRM-area types are not significantly different than health outcome trends in non-CBNRM areas.

To test the robustness of our results we calculate differences-in-differences estimates using only households that directly intersect the census tract of villages involved in CBNRM (0 km distance). Our wealth and food security results are robust when using this sample (see S8 Table). In fact, the difference-in-differences coefficients are stronger in magnitude and more significant than the 5 km distance sample as there are likely fewer non-CBNRM households diluting the CBNRM sample (and subsequently the CBNRM effect). However, height-age and weight-age measures are not robust to the restricted (0 km distance) sample; the weakly significantly positive impact of JFM areas on temporal changes in height-age and weight-age ratios found using the 5 km buffer sample are no longer significant in the 0 km distance sample.

### Wealth Model

The triple differences model examines the differential effect of CBNRM on observations with average or above average wealth (wealth indexes greater than or equal to zero) compared with observations with below average wealth (negative wealth indexes). These results, presented in Table 9, show that wealthy households appear to benefit more from CBNRM than poor households. The number of meals per day increased significantly more in wealthy households in JFM and CBFM areas over the study period compared with wealthy households outside CBNRM areas. The change in number of meals consumed per day in JFM areas and the change in meat/fish consumption in poor households in CBFM areas is significantly lower than in poor households in non-CBNRM areas. Both wealthy and poor households in WMAs experienced improvements in food security outcomes. The increase in problems meeting food needs was significantly lower for both wealthy and poor households in WMAs compared with households outside WMAs. In addition, poor households in WMAs increased meat and fish consumption significantly more than poor, non-CBNRM households between 2003 and 2012.

**Table 9:** Differential effects of CBNRM on positive and negative wealth indexes.

	Households with wealth index > = 0			Households with wealth index <0		
	JFM	CBFM	WMA	JFM	CBFM	WMA
Meals/day	0.220**	0.112**	0.0105	-0.215**	-0.0272	0.0298
	(0.0923)	(0.0542)	(0.0731)	(0.0897)	(0.0531)	(0.0685)
Meat/fish per week	-0.0370	0.0160	-0.0127	0.0468	-0.0291*	0.0559***
	(0.0239)	(0.0148)	(0.0198)	(0.0297)	(0.0150)	(0.0200)
Problems satisfying food needs last year	-0.0356	-0.0646	-0.395***	-0.137	-0.129	-0.230*
	(0.148)	(0.0988)	(0.127)	(0.146)	(0.0997)	(0.122)
Weight/age	0.4952**	0.1951	0.1502	-0.3065*	-0.05065	0.1520
	(0.2134)	(0.1388)	(0.1916)	(0.1859)	(0.1117)	(0.1594)
Height/age	0.5658*	0.2610	0.05587	-0.3359	-0.08603	0.1260
	(0.2979)	(0.1784)	(0.1913)	(0.2839)	(0.1517)	(0.1838)
Weight/height	0.2644	0.02653	0.1199	-0.2085	0.0305	0.1153
	(0.2425)	(0.1487)	(0.2002)	(0.2196)	(0.1282)	(0.1949)

This table shows the triple differences estimates for each of the CBNRM types on each dependent variable for observations, isolating the effects on observations with wealth indexes greater than or equal to zero versus wealth indexes less than zero. The wealth index > = 0 estimate is the CBNRM*year interaction term coefficient. The wealth index <0 estimate is the CBNRM*year*negativewealthindex interaction term, which captures the differential effect of CBNRM on poor households in CBNRM areas. Robust standard errors are in parentheses.

*** indicates significance at the 1% level,

** at the 5% level, and

* at the 10% level.

Wealthy households in JFM areas have significantly larger changes in weight-age and height-age ratios, indicating that both in the short-run (weight-age ratio) and long-run (height-age ratio) children from wealthy families in JFM areas are experiencing increasingly better health outcomes than wealthy children outside JFM areas. However, the change in weight-age ratios in poor households in JFM areas between 2003 and 2012 is significantly lower than poor households outside JFM areas. These results are consistent with our findings that meal consumption in poor households in JFM areas is significantly lower over time than in poor households outside CBNRM areas. Weight-age ratios change with changes in caloric intake, so it is not surprising that a group consuming comparatively fewer meals over time also has comparatively lower weight-age ratios over time.

### Timing of CBNRM Establishment

Our final model examines whether CBNRM activities initiated prior to 2005 have a stronger positive effect on households than CBNRM activities initiated in 2005 or later. Results in Table 10 show the advantages of longer-standing CBNRM activities. Households in CBFM areas initiated prior to 2005 experienced a significantly greater increase in the number of meals consumed per day between 2003 and 2012 compared with households in non-CBNRM areas. However, the change in the number of meals consumed per day is significantly lower in CBFM households initiated in 2005 or later. Households in WMA areas initiated prior to 2005 experience a significantly lower increase in problems satisfying food needs between 2003 and 2012 compared with non-CBNRM areas. The trends in problems satisfying food needs for households in WMAs initiated after 2005 are not significantly different than in households outside CBNRM areas. In addition, the change in meat/fish consumption in recently-established WMAs is significantly lower than in non-CBNRM areas over the study period.

**Table 10:** Differential effects of CBNRM established pre and post-2005.

	Pre-2005 effect			Post-2005 effect		
	JFM	CBFM	WMA	JFM	CBFM	WMA
Wealth index	-0.00721	-0.0171	-0.0463	-0.153	0.0372	0.115
	(-0.0607)	(-0.0414)	(-0.079)	(-0.153)	(-0.0674)	(-0.137)
Meals/day	0.0476	0.138***	0.0316	-0.213	-0.170**	0.0163
	(-0.0683)	(-0.0394)	(-0.0577)	(-0.165)	(-0.0731)	(-0.184)
Meat/fish per week	-0.0146	0.00392	0.0309	0.0796	0.00229	-0.164***
	(-0.0192)	(-0.0149)	(-0.0194)	(-0.0535)	(-0.0261)	(-0.0591)
Problems satisfying food needs last year	-0.129	-0.119	-0.555***	0.00116	-0.11	-0.0256
	(-0.113)	(-0.0804)	(-0.111)	(-0.274)	(-0.14)	(-0.37)
Weight/age	0.1253	0.08434	0.1484	0.2928	0.2858	0.167
	(-0.1864)	(-0.1805)	(-0.2312)	(-0.4392)	(-0.2599)	(-0.387)
Height/age	0.3494	0.0607	0.1663	-0.5004	0.09527	-0.4744
	(-0.2134)	(-0.2001)	(-0.2551)	(-0.4166)	(-0.3329)	(-0.4034)
Weight/height	-0.07508	-0.198	0.07377	0.6451	0.29	0.4145
	(-0.2004)	(-0.1859)	(-0.1939)	(-0.4382)	(-0.2665)	(-0.4119)

This table shows different effects of wealth, food security and health outcomes based on CBNRM initiation year. Estimates for CBNRM area effects prior to 2005 are the CBNRM*year interaction term coefficients. Estimates for CBNRM effects in areas initiated in 2005 and later are the CBNRM*year*post2005 interaction term, which captures the differential effect of CBNRM areas established in or after 2005. Robust standard errors are in parentheses.

*** indicates significance at the 1% level and

** at the 5% level.

## Discussion

Devolving natural resources governance to local communities through CBNRM can improve stewardship of natural resources and increase community members’ access to resource benefits [1,2]. In turn, this may improve community members’ livelihoods and welfare. Our results show that trends in wealth indexes in JFM and WMA households between 2003 and 2012 are not significantly different than trends in wealth indexes in non-CBNRM areas during the same period. In the case of CBFM, wealth index increased at a significantly lower rate than non-CBNRM households since CBNRM implementation. Trends in household food security measures appear to be, for the most part, better in CBNRM areas than non-CBNRM areas, but trends in health outcomes are generally not significantly different for children in CBNRM areas.

There do appear to be differential effects of CBNRM on wealthy and poor households in CBFM and JFM areas. Trends in food security indicators for wealthy households tend to be better in JFM and CBFM areas and worse for poor households. Results for both long and short-term health indicators in children under 5 years in JFM areas support this. Wealthy children in JFM areas have significantly higher height and weight-age ratios over time; poor children, in contrast, have lower weight-age ratios over time. These results are consistent with the finding that changes in meal consumption are lower in JFM households compared with non-CBNRM households. Children from less food secure (poor) households demonstrate lower growth rates over time than children from more food secure (wealthy) households in JFM areas. WMAs do not demonstrate differential wealth effects, and significantly increase food security for both wealthy and poor households compared with non-CBNRM households over the study period.

Evidence for other intended impacts of community conservation, i.e., improved forest and wildlife management, is mixed, but tends to be positive when available. For example Blomley et al. [19] and Persha and Blomley [23] show a number of broadly positive outcomes for forest condition from CBFM and JFM. Neilsen and Treue [49] find reduced hunting pressures and subsequent wildlife recovery in JFM areas. In some WMAs there is also evidence of positive wildlife trends, especially in the northern WMAs around larger state reserves [20].

The lack of a positive effect of CBNRM on wealth is consistent with other reports of poverty impacts of CBNRM in Tanzania [23,26]. Economic opportunities from CBNRM are limited, largely due to restrictions on resource use and an inability to generate sufficient revenues to contribute to household incomes [20,22]. CBFM and JFM sites tend to be too small to have significant revenue from forest products [23]. Benefit sharing among multiple communities can also reduce income benefits. For instance, Minjingu Village's revenues declined when it joined a WMA because funds are shared equally among participating villages [20].

WMAs were additionally influenced by modifications to Tanzania’s wildlife policy in 2007 and introduction of the Non-Consumptive Utilization of Wildlife Regulations in 2008 [21]. These policies recentralized wildlife management, which not only caused donors to withdraw support for WMAs, but also required a portion of fees from non-consumptive wildlife use to go to the central government, ultimately reducing WMA-generated income [21]. Indeed our results show evidence of the effects of these policies; WMA households enjoy increasing wealth through 2007, and then experience a sharp decline after 2007 (Fig 2). Although wealth measures themselves may not have improved for CBNRM households, it is important to consider other potential contributions of CBNRM areas to household-level economic diversification, improved response to economic shocks and overall well-being, in addition to community-level benefits such as improved infrastructure and public services [2,27].

Our results show some evidence that CBNRM provides food security benefits. The number of meals consumed per day increased in JFM and CBFM areas between 2003 and 2012, yet decreased in non-CBNRM areas. Households in CBFM and WMA areas also experienced a significantly lower increase in problems meeting food needs over the study period than non-CBNRM households. Improved access to natural resources and food sources through CBNRM could help to smooth fluctuations in food access, explaining why we see a decline in meals consumed per day and increased frequency meeting food needs outside CBNRM areas, but less so within CBNRM areas.

Although food security measures generally improved in CBNRM areas, meat/fish consumption in JFM areas appears to be negatively affected. Prior to CBNRM implementation, households in JFM areas consumed meat and fish more often than households in non-CBNRM areas. Between 2003 and 2012 meat and fish consumption increased substantially in non-CBNRM households, but did not change in JFM households. This difference in trends in meat/fish consumption between non-CBNRM and JFM households suggests that JFM implementation had a negative effect on meat/fish consumption. However this effect could be due to extraordinarily high meat/fish consumption in JFM households from the outset, rather than an adverse effect of JFM.

In general, changes in health outcomes in children under five are no different in CBNRM areas than outside CBNRM areas. Children in JFM areas have slightly higher changes in height-age and weight-age ratios, but these are weakly significant (p<10%) and are not robust when using the sample that directly intersects CBNRM village census tracts (see S8 Table). That health outcomes in children are generally not significantly different inside and outside CBNRM areas is not entirely surprising. Even if food security outcomes have improved, it takes time for these benefits to translate to measurable health outcomes. In addition, child health depends on not only household wealth and food security, but also intra-household resource allocation. In other words, even if household food security increases, this does not mean that children will be better off since additional resources will not necessarily be allocated to children.

CBNRM does not appear to improve household wealth, but does improve food security. This combination of results is somewhat surprising given wealth and food security are positively related [43]. The wealth index is constructed using information on household assets, house construction materials and access to public services such as water and markets (S1 Text). Wealth measures may not be as responsive to changes in resource access as food security measures. Households may have improved access to food sources through CBNRM, directly increasing food security. Wealth measures may be more responsive to improvements to the natural resource base than access to it, therefore it could take years to see measurable improvements in forest or wildlife conditions and any subsequent effect on wealth.

That CBNRM areas yield more food security benefits (short-term) than wealth and health benefits (long-term) suggests the timing of CBNRM implementation can influence its observable benefits. Our results comparing CBNRM areas established pre and post-2005 support this. CBNRM areas initiated prior to 2005 show significantly better food security outcomes than those initiated after 2005, suggesting that it takes time for CBNRM activities to provide measurable benefits. These results mirror CBNRM studies that find CBNRM benefits increase with longevity [19,47]. Sufficient time may not have elapsed since CBNRM implementation to see substantial improvements in the natural resource base (see [19]) and consequently in its benefits to communities.

Although the three CBNRM types we examined (CBFM, JFM and WMAs) exhibit important differences in the level of community governance, structure and location characteristics, our results do not provide clear evidence that any one type of CBNRM is more effective at improving welfare than the others. We expected wealth, food security and health outcomes to vary considerably across the three different CBNRM types. Since JFM sites are not fully community-owned, have poorly defined tenure, higher restrictions and limited access to resources when compared with CBFM areas or WMAs [15,26], we expected them to not do as well in the measured indicators. Our results, however, show that they have increased meals/day and although meat/fish consumption declined, this seems to be more a function of relatively high meat/fish consumption from the outset. Likewise, since WMAs are large and provide tourism opportunities through wildlife resources [4,20,50], we would expect them to do even better than CBFM and JFM areas, which are substantially smaller and do not have the same tourism opportunities. However, WMAs do not appear to be better off than the other CBNRM types, perhaps due to the deleterious wildlife policy changes in the mid-2000s.

One explanation for the lack of observed variation between CBNRM types is the limited amount of time for implementation. Establishment of JFMs, CBFMs and WMAs takes years, and tangible improvements in forest and wildlife resources take even longer [19]. Since most CBNRM areas have been established within the past decade, it is possible that larger improvements in wealth, food security and health outcomes will be seen in the future and some areas (or types of areas) will prove to be more effective than others. In addition, only a fraction of CBNRM agreements are currently formally recognized. Perhaps as more are formalized we will continue to see increased community benefits and greater differences among CBNRM types. Finally, some scholars have found that CBNRM activity tends to take place on low-quality lands [15,51]. If resources under CBNRM are of low quality, then the specific governance regime may have little influence on CBNRM benefits. Perhaps we would see more variation in the different CBNRM types if the tenure arrangements were protecting higher-value resources.

## Conclusions

Here we compile and explore a cross section of national-scale data across nearly a decade to identify welfare benefits of CBNRM. In doing so we present some evidence of positive food security benefits of CBNRM, additional benefits of longer-standing CBNRM activity and a differential wealth effect. We do not detect much evidence of wealth or health outcomes, or of differences in effects of the three CBNRM governance regimes. Whether or not this is because CBNRM hasn’t been around long enough to yield demonstrable outcomes, or because there simply is little effect of CBNRM on welfare is difficult to determine. Limited evidence of CBNRM welfare impacts may also be a function of available data. Although we are able to compile a cross section of household data through the 2000s, with panel data we could track individual households over time to identify within-household variation attributable to CBNRM. In addition, while village census tract locations are available, our analysis would be strengthened with precise CBNRM locations; however these data do not yet exist.

We do not assess whether or not the benefits of CBNRM outweigh the costs, yet CBNRM’s benefits to rural populations will be critical for its success. CBNRM is costly, and if the benefits are insufficient, then communities will not have incentives to continue participation, losing the potential ecological and socioeconomic benefits that CBNRM has to offer. REDD+ and Payments for Ecosystem Services (PES) programs are mechanisms that might improve benefits to CBNRM villages [22]. CBNRM communities are already self-organized and implementing sustainable resource management plans; the costs of establishing REDD+ and PES programs are thus greatly reduced [5,7–9,50]. Offering CBNRM communities payments for their efforts through REDD+ or PES may provide them with additional benefits to continue sustainable resource management efforts.

## Supporting Information

S1 Text Wealth Index Defined. Excerpt from Page 39 Tanzania HIV/AIDS and Malaria Indicator Survey 2011–12.
(DOCX)

S1 Table Complete difference-in-differences model for dependent variable: wealth index. This table shows full results of difference-in-differences models for JFM, CBFM and WMA, including all control variables. *** p<0.01, ** p<0.05, * p<0.1.
(DOCX)

S2 Table Complete difference-in-differences model for dependent variable: meals/day. This table shows full results of difference-in-differences models for JFM, CBFM and WMA, including all control variables. *** p<0.01, ** p<0.05, * p<0.1.
(DOCX)

S3 Table Complete difference-in-differences model for dependent variable: meat/fish/week. This table shows full results of difference-in-differences models for JFM, CBFM and WMA, including all control variables. *** p<0.01, ** p<0.05, * p<0.1.
(DOCX)

S4 Table Complete difference-in-differences model for dependent variable: problems satisfying food needs. This table shows full results of difference-in-differences models for JFM, CBFM and WMA, including all control variables. *** p<0.01, ** p<0.05, * p<0.1.
(DOCX)

S5 Table Complete difference-in-differences model for dependent variable: weight/age Z-score. This table shows full results of difference-in-differences models for JFM, CBFM and WMA, including all control variables. *** p<0.01, ** p<0.05, * p<0.1.
(DOCX)

S6 Table Complete difference-in-differences model for dependent variable: height/age Z-score. This table shows full results of difference-in-differences models for JFM, CBFM and WMA, including all control variables. *** p<0.01, ** p<0.05, * p<0.1.
(DOCX)

S7 Table Complete difference-in-differences model for dependent variable: weight/height Z-score. This table shows full results of difference-in-differences models for JFM, CBFM and WMA, including all control variables. *** p<0.01, ** p<0.05, * p<0.1.
(DOCX)

S8 Table Differences-in-differences model results for wealth, food security and health outcomes using a sample of households that directly intersect the CBNRM village census tracts (i.e., no 5 km buffer). This table shows full results of difference-in-differences models for JFM, CBFM and WMA, including all control variables for those households that directly intersect the CBNRM village census tracts. *** p<0.01, ** p<0.05, * p<0.1.
(DOCX)

## References

[1] Ostrom E. Governing the Commons: The Evolution of Institutions for Collective Action. Cambridge, UK: Cambridge University Press; 1990.

[2] Lund JF. Is small beautiful? Village level taxation of natural resources in Tanzania. Public Adm Dev. 2007;27: 307–318. 10.1002/pad.467

[3] Agrawal A, Ostrom E. Collective Action, Property Rights, and Decentralization in Resource Use in India and Nepal. Polit Soc. 2001;29: 485–514. 10.1177/0032329201029004002

[4] Persha L, Agrawal A, Chhatre A. Social and ecological synergy: local rulemaking, forest livelihoods, and biodiversity conservation. Science. 2011;331: 1606–8. 10.1126/science.1199343 21436453

[5] Bowler D, Buyung-Ali L, Healey JR, Jones JPG, Knight T, Pullin AS. The evidence base for community forest management as a mechanism for supplying global environmental benefits and improving local welfare. Centre for Evidence-Based Conservation, Bangor University. Bangor, Gwynedd, UK; 2010. Report No.: CEE review 08–011. Available: http://citeseerx.ist.psu.edu/viewdoc/download?rep=rep1&type=pdf&doi=10.1.1.220.6153

[6] Agrawal A, Chhatre A, Hardin R. Changing governance of the world’s forests. Science. 2008;320: 1460–2. 10.1126/science.1155369 18556552

[7] Burgess ND, Bahane B, Clairs T, Danielsen F, Dalsgaard S, Funder M, et al Getting ready for REDD+ in Tanzania: a case study of progress and challenges. Oryx. 2010;44: 339–351. 10.1017/S0030605310000554

[8] Danielsen F, Adrian T, Brofeldt S, van Noordwijk M, Poulsen MK, Rahayu S, et al Community Monitoring for REDD+: International Promises and Field Realities. Ecol Soc. 2013;18 10.5751/ES-05464-180341

[9] Robinson EJZ, Albers HJ, Meshack C, Lokina RB. Implementing REDD through community-based forest management: Lessons from Tanzania. Nat Resour Forum. 2013;37: 141–152. 10.1111/1477-8947.12018

[10] Andam KS, Ferraro PJ, Pfaff A, Sanchez-Azofeifa GA, Robalino JA. Measuring the effectiveness of protected area networks in reducing deforestation. Proc Natl Acad Sci U S A. 2008;105: 16089–94. 10.1073/pnas.0800437105 18854414 PMC2567237

[11] Joppa LN, Pfaff A. High and far: biases in the location of protected areas. PLoS One. 2009;4: e8273 10.1371/journal.pone.0008273 20011603 PMC2788247

[12] Andam KS, Ferraro PJ, Sims KRE, Healy A, Holland MB. Protected areas reduced poverty in Costa Rica and Thailand. Proc Natl Acad Sci U S A. 2010;107: 9996–10001. 10.1073/pnas.0914177107 20498058 PMC2890456

[13] Sims KRE. Conservation and development: Evidence from Thai protected areas. J Environ Econ Manage. 2010;60: 94–114. 10.1016/j.jeem.2010.05.003

[14] Pfaff A, Robalino J, Lima E, Sandoval C, Herrera LD. Governance, Location and Avoided Deforestation from Protected Areas: Greater Restrictions Can Have Lower Impact, Due to Differences in Location. World Dev. 2014;55: 7–20. 10.1016/j.worlddev.2013.01.011

[15] Blomley T, Iddi S. Participatory Forest Management in Tanzania 1993–2009: Lessons Learned and experiences to date. 2009.

[16] Ferraro PJ, Hanauer MM. Quantifying causal mechanisms to determine how protected areas affect poverty through changes in ecosystem services and infrastructure. Proc Natl Acad Sci U S A. 2014;111: 4332–7. 10.1073/pnas.1307712111 24567397 PMC3964111

[17] Kennedy P. A Guide to Econometrics. 5th ed Cambridge, MA: MIT Press; 2003.

[18] Angrist JD, Pischke J-S. Mostly Harmless Econometrics: An Empiricist’s Companion. 1st ed Princeton, NJ: Princeton University Press; 2009.

[19] Blomley T, Pfliegner K, Isango J, Zahabu E, Ahrends A, Burgess N. Seeing the wood for the trees: an assessment of the impact of participatory forest management on forest condition in Tanzania. Oryx. 2008;42: 380–391. 10.1017/S0030605308071433

[20] World Wildlife Fund (WWF). Tanzania’ s Wildlife Management Areas A 2012 Status Report. Dar es Salaam, Tanzania; 2014.

[21] Benjaminsen TA, Goldman MJ, Minwary MY, Maganga FP. Wildlife Management in Tanzania: State Control, Rent Seeking and Community Resistance. Dev Change. 2013;44: 1087–1109. 10.1111/dech.12055

[22] Ministry of Natural Resources and Tourism (MNRT). Participatory Forest Management in Tanania: Facts and Figures. Dar es Salaam, Tanzania; 2012.

[23] Persha L, Blomley T. Management decentralization and montane forest conditions in Tanzania. Conserv Biol. 2009;23: 1485–96. 10.1111/j.1523-1739.2009.01276.x 19558523

[24] Treue T, Ngaga YM, Meilby H, Lund JF, Kajembe G, Iddi S, et al Does participatory forest management promote sustainable forest utilisation in Tanzania? Int For Rev. Commonwealth Forestry Association; 2014;16: 23–38. 10.1505/146554814811031279

[25] Runsten L, Ravilious C, Kashindye A, Giliba R, Hailakwahi V, Kashaga LRA, et al. Using spatial information to support decisions on safeguards and multiple benefits for REDD+ in Tanzania. Dar es Salaam, Tanzania; 2013.

[26] Ministry of Natural Resources and Tourism (MNRT). Selected case studies from the eastern arc mountains area of Tanzania: Action Research into Poverty Impacts of Participatory Forest Management, Conservation and Management of Eastern Arc Mountains Forests. Dar es Salaam; 2009.

[27] Ribot JC, Lund JF, Treue T. Democratic decentralization in sub-Saharan Africa: its contribution to forest management, livelihoods, and enfranchisement. Environ Conserv. 2010;37: 35–44. 10.1017/S0376892910000329

[28] Tanzania Commission for AIDS (TACAIDS), (NBS) National Bureau of Statistics, ORC Macro. Tanzania HIV/AIDS Indicator Survey 2003–04. 2005.

[29] Tanzania Commission for AIDS (TACAIDS), Zanzibar AIDS Commission, (NBS) National Bureau of Statistics, Office of the Chief Government Statistician (OCGS), Inc MI. Tanzania HIV/AIDS and Malaria Indicator Survey 2007–08. Dar es Salaam, Tanzania; 2008.

[30] Tanzania Commission for AIDS (TACAIDS), Zanzibar AIDS Commission, (NBS) National Bureau of Statistics [Tanzania], Office of the Chief Government Statistician (OCGS), ICF International. Tanzania HIV/AIDS and Malaria Indicator Survey 2011–12. Dar es Salaam, Tanzania; 2013.

[31] National Bureau of Statistics (NBS) [Tanzania] and Macro International Inc. Tanzania Reproductive and Child Health Survey 1999. Dar es Salaam, Tanzania; 2000.

[32] National Bureau of Statistics (NBS) [Tanzania], ICF Macro. Tanzania Demographic and Health Survey 2010. Dar es Salaam, Tanzania; 2011.

[33] Trabucco A, Zomer RJ. Global Aridity Index (Global-Aridity) and Global Potential Evapo-Transpiration (Global-PET) Geospatial Database [Internet]. Global Aridity Index (Global-Aridity) and Global Potential Evapo-Transpiration (Global-PET) Geospatial Database. 2009. Available: Published online, available from the CGIAR-CSI GeoPortal at: http://www.csi.cgiar.org

[34] Kariuki PC. Tanzania Landuse 2002 [Internet]. International Livestock Research Institute. 2007. Available: http://192.156.137.110/gis/default.asp

[35] UCN, UNEP-WCMC. The World Database on Protected Areas (WDPA) [On-line] [Internet]. Cambridge, UK: UNEP-WCMC; 2014. Available: www.protectedplanet.net

[36] National Bureau of Statistics (NBS), Ministry of Planning Economy and Empowerment [Tanzania]. Tanzania Population and Housing Census [Internet]. Dar es Salaam, Tanzania; 2002. Available: www.nbs.go.tz

[37] Rutstein SO, Johnston K. The DHS Wealth Index. DHS Comparative Reports No. 6. Calverton, MD; 2004.

[38] WHO Multicentre Growth Reference Study Group. WHO Child Growth Standards: Length/height-for-age, weight-for-age, weight-for-length, weight-for-height and body mass index-for-age: Methods and development. Geneva; 2006.

[39] IFAD. Investing in rural people in the United Republic of Tanzania [Internet]. Rome, Italy; 2014. Available: http://www.ifad.org/operations/projects/regions/pf/factsheets/tanzania.pdf

[40] Mackenzie CA. Accruing benefit or loss from a protected area: Location matters. Ecol Econ. 2012;76: 119–129. 10.1016/j.ecolecon.2012.02.013

[41] Baird TD. Conservation and Unscripted Development : Proximity to Park Associated with Development and Financial Diversity. Ecol Soc. 2014;19 10.5751/ES-06184-190104

[42] Schaafsma M. Mapping NTFP collection in Tanzania: A comparison of surveys [Internet]. Norwich, UK; 2012. Report No.: 2012–05. Available: http://www.cserge.ac.uk/sites/default/files/2012-05.pdf

[43] Kennedy E, Peters P. Household food security and child nutrition: the interaction of income and gender of household head. World Dev. 1992;20: 1077–1085. 10.1016/0305-750X(92)90001-C

[44] Meshack CK, Ahdikari B, Doggart N, Lovett JC. Transaction costs of community-based forest management: empirical evidence from Tanzania. Afr J Ecol. 2006;44: 468–477. 10.1111/j.1365-2028.2006.00659.x

[45] Lund JF, Treue T. Are We Getting There? Evidence of Decentralized Forest Management from the Tanzanian Miombo Woodlands. World Dev. 2008;36: 2780–2800. 10.1016/j.worlddev.2008.01.014

[46] Vyamana VG, Chonya AB, Sasu F V, Rilagonya F, Gwassa FN, Kivamba S. Participatory Forest Management in the Eastern Arc Mountain area of Tanzania : Who is benefiting ? Int For Rev. 2009;11: 293–253.

[47] Naidoo R, Stuart-Hill G, Weaver LC, Tagg J, Davis A, Davidson A. Effect of Diversity of Large Wildlife Species on Financial Benefits to Local Communities in Northwest Namibia. Environ Resour Econ. 2010;48: 321–335. 10.1007/s10640-010-9412-3

[48] Schreckenberg K, Luttrell C. Participatory forest management: a route to poverty reduction? Int For Rev. Commonwealth Forestry Association; 2009;11: 221–238. 10.1505/ifor.11.2.221

[49] Nielsen MR, Treue T. Hunting for the Benefits of Joint Forest Management in the Eastern Afromontane Biodiversity Hotspot: Effects on Bushmeat Hunters and Wildlife in the Udzungwa Mountains. World Dev. 2012;40: 1224–1239. 10.1016/j.worlddev.2011.11.009

[50] Chhatre A, Agrawal A. Trade-offs and synergies between carbon storage and livelihood benefits from forest commons. Proc Natl Acad Sci U S A. 2009;106: 17667–70. 10.1073/pnas.0905308106 19815522 PMC2764886

[51] Mustalahti I, Lund JF. Where and How Can Participatory Forest Management Succeed? Learning From Tanzania, Mozambique, and Laos. Soc Nat Resour; 2009;23: 31–44. 10.1080/08941920802213433

