# Supplementary material for: Impacts of Community-Based Natural Resource Management on Wealth, Food Security and Child Health in Tanzania
Source: PLoS One. 2015 Jul 17;10(7):e0133252. doi: 10.1371/journal.pone.0133252 (PMC4506085; doi:10.1371/journal.pone.0133252)
Supplement: S1 Text — Excerpt from Page 39 Tanzania HIV/AIDS and Malaria Indicator Survey 2011–12. (DOCX) [file pone.0133252.s001.docx]

**S1. Wealth Index Defined**: Excerpt from Page 39 Tanzania HIV/AIDS and Malaria Indicator Survey 2011-12

“The wealth index is constructed using household asset data, including ownership of consumer items ranging from a television to a bicycle or car, as well as dwelling characteristics, such as source of drinking water, sanitation facilities, and type of flooring material. In its current form, which takes account of urban-rural differences in these items and characteristics, the wealth index is created in three steps. In the first step, a subset of indicators common to urban and rural areas is used to create wealth scores for households in both areas. For purposes of creating scores, categorical variables are transformed into separate dichotomous (0-1) indicators. These indicators and those that are continuous are then examined using principal components analysis to produce a common factor score for each household. In the second step, separate factor scores are produced for households in urban and rural areas using area-specific indicators (Rutstein, 2008). The third step combines the separate area-specific factor scores to produce a nationally applicable combined wealth index by adjusting area-specific scores through a regression on the common factor scores. The resulting combined wealth index has a mean of zero and a standard deviation of one.”

“This method for calculating a country-specific wealth index was developed and tested in a large number of countries in relation to inequalities in household income, use of health services, and health outcomes (Rutstein and Johnson, 2004). It has been shown to be consistent with expenditure and income measures.”

Rutstein, S., and K. Johnson. 2004. *The DHS Wealth Index*. DHS Comparative Reports No. 6.

Calverton, Maryland: ORC Macro.

Rutstein, S. 2008. *The DHS Wealth Index: Approaches for Rural and Urban Areas*. DHS Working Papers. Calverton, Maryland: Macro International.

Tanzania Commission for AIDS (TACAIDS), Zanzibar AIDS Commission (ZAC), National Bureau of Statistics (NBS), Office of the Chief Government Statistician (OCGS), and ICF International 2013. *Tanzania HIV/AIDS and Malaria Indicator Survey 2011-12.* Dar es Salaam, Tanzania: TACAIDS, ZAC, NBS, OCGS, and ICF International.
