# Supplementary material for: Impacts of Community-Based Natural Resource Management on Wealth, Food Security and Child Health in Tanzania
Source: PLoS One. 2015 Jul 17;10(7):e0133252. doi: 10.1371/journal.pone.0133252 (PMC4506085; doi:10.1371/journal.pone.0133252)
Supplement: S1 Table — This table shows full results of difference-in-differences models for JFM, CBFM and WMA, including all control variables. *** p<0.01, ** p<0.05, * p<0.1. (DOCX) [file pone.0133252.s002.docx]

**S2. Complete Difference-in-differences model for dependent variable: wealth index**

| VARIABLES | JFM | CBFM | WMA |
| --- | --- | --- | --- |
| Number household members | 0.0134*** | 0.0131*** | 0.0135*** |
|  | (0.00229) | (0.00218) | (0.00232) |
| Number children under 5 | -0.0717*** | -0.0690*** | -0.0718*** |
|  | (0.00546) | (0.00519) | (0.00556) |
| Max number years education* | 0.0704*** | 0.0727*** | 0.0697*** |
|  | (0.00221) | (0.00212) | (0.00225) |
| Single adult head of hh | 0.0872*** | 0.0857*** | 0.0744*** |
|  | (0.0162) | (0.0154) | (0.0165) |
| Female head of hh | -0.0974*** | -0.0911*** | -0.101*** |
|  | (0.0128) | (0.0121) | (0.0130) |
| Regional Avg 1999 Wealth | 0.0208 | 0.0289 | 0.0766*** |
|  | (0.0300) | (0.0273) | (0.0297) |
| Within 5km Protected Area | 0.0960*** | 0.0988*** | 0.0848*** |
|  | (0.0110) | (0.0103) | (0.0112) |
| Within 5km Forest Reserve | -0.0332*** | -0.0371*** | -0.0380*** |
|  | (0.0112) | (0.0105) | (0.0113) |
| Urban | 0.958*** | 0.955*** | 0.969*** |
|  | (0.0213) | (0.0211) | (0.0220) |
| Nearest Market (km) | -0.00278*** | -0.00248*** | -0.00271*** |
|  | (0.000215) | (0.000197) | (0.000216) |
| Central Region | -0.166*** | -0.143*** | -0.167*** |
|  | (0.0307) | (0.0299) | (0.0311) |
| South Region | -0.415*** | -0.385*** | -0.419*** |
|  | (0.0261) | (0.0256) | (0.0267) |
| SW Highlands Region | -0.205*** | -0.148*** | -0.203*** |
|  | (0.0300) | (0.0288) | (0.0300) |
| Lake Region | -0.187*** | -0.162*** | -0.176*** |
|  | (0.0276) | (0.0272) | (0.0281) |
| West Region | -0.242*** | -0.201*** | -0.229*** |
|  | (0.0292) | (0.0285) | (0.0294) |
| North Region | 0.0819** | 0.0708** | 0.0413 |
|  | (0.0345) | (0.0328) | (0.0347) |
| South Highlands Region | -0.103*** | -0.0759*** | -0.107*** |
|  | (0.0288) | (0.0280) | (0.0283) |
| Percent bushland | -0.283*** | -0.294*** | -0.218*** |
|  | (0.0407) | (0.0404) | (0.0396) |
| Percent cultivated land | -0.213*** | -0.222*** | -0.135*** |
|  | (0.0405) | (0.0402) | (0.0392) |
| Percent grassland | -0.326*** | -0.317*** | -0.262*** |
|  | (0.0425) | (0.0420) | (0.0415) |
| Percent woodland | -0.423*** | -0.421*** | -0.359*** |
|  | (0.0416) | (0.0410) | (0.0404) |
| Percent natural forest | -0.468*** | -0.405*** | -0.353*** |
|  | (0.0649) | (0.0651) | (0.0715) |
| District-level population density | 0.000164*** | 0.000166*** | 0.000148*** |
|  | (1.24e-05) | (1.23e-05) | (1.20e-05) |
| Percent economically active population | 1.302*** | 1.421*** | 1.546*** |
|  | (0.392) | (0.390) | (0.404) |
| Percent voting population | 0.107 | 0.0396 | -0.127 |
|  | (0.394) | (0.392) | (0.406) |
| Elevation | 9.61e-05*** | 7.99e-05*** | 8.02e-05*** |
|  | (1.67e-05) | (1.60e-05) | (1.70e-05) |
| Slope | 0.0113*** | 0.0133*** | 0.0113*** |
|  | (0.00232) | (0.00221) | (0.00243) |
| Aridity Index | 5.11e-07 | -2.97e-07 | -6.00e-08 |
|  | (3.32e-06) | (3.20e-06) | (3.37e-06) |
| 2007 | 0.170*** | 0.164*** | 0.165*** |
|  | (0.0141) | (0.0139) | (0.0141) |
| 2012 | 0.0904*** | 0.0864*** | 0.0895*** |
|  | (0.0125) | (0.0123) | (0.0125) |
| CBNRM dummy | -0.199*** | 0.0286 | -0.136** |
|  | (0.0379) | (0.0238) | (0.0554) |
| CBNRM*2007 | 0.00847 | -0.156*** | 0.193** |
|  | (0.0519) | (0.0318) | (0.0755) |
| CBNRM*2012 | 0.0675 | -0.0624** | -0.0278 |
|  | (0.0481) | (0.0294) | (0.0702) |
| Constant | -1.303*** | -1.359*** | -1.340*** |
|  | (0.0874) | (0.0863) | (0.0881) |
|  |  |  |  |
| Observations | 16,753 | 18,268 | 16,614 |
| R-squared | 0.512 | 0.507 | 0.520 |
| Robust standard errors in parentheses  *** p<0.01, ** p<0.05, * p<0.1 |  |  |  |
|  |  |  |  |
