# Supplementary material for: Impacts of Community-Based Natural Resource Management on Wealth, Food Security and Child Health in Tanzania
Source: PLoS One. 2015 Jul 17;10(7):e0133252. doi: 10.1371/journal.pone.0133252 (PMC4506085; doi:10.1371/journal.pone.0133252)
Supplement: S2 Table — This table shows full results of difference-in-differences models for JFM, CBFM and WMA, including all control variables. *** p<0.01, ** p<0.05, * p<0.1. (DOCX) [file pone.0133252.s003.docx]

**S3. Complete Difference-in-differences model for dependent variable: meals/day**

| VARIABLES | JFM | CBFM | WMA |
| --- | --- | --- | --- |
| Wealth Index | -0.234*** | -0.236*** | -0.234*** |
|  | (0.00693) | (0.00670) | (0.00681) |
| Number household members | 0.00995*** | 0.00936*** | 0.00959*** |
|  | (0.00197) | (0.00187) | (0.00197) |
| Number children under 5 | -0.0253*** | -0.0241*** | -0.0226*** |
|  | (0.00503) | (0.00478) | (0.00503) |
| Max number years education* | -0.0120*** | -0.0121*** | -0.0130*** |
|  | (0.00183) | (0.00174) | (0.00182) |
| Single adult head of hh | -0.00776 | -0.00428 | -0.00714 |
|  | (0.0146) | (0.0139) | (0.0145) |
| Female head of hh | 0.0895*** | 0.0832*** | 0.0942*** |
|  | (0.0109) | (0.0104) | (0.0109) |
| Regional Avg 1999 Wealth | -0.0462* | -0.0172 | -0.0330 |
|  | (0.0243) | (0.0228) | (0.0235) |
| Within 5km Protected Area | 0.0135 | 0.00930 | 0.0105 |
|  | (0.00972) | (0.00918) | (0.00981) |
| Within 5km Forest Reserve | -0.0276*** | -0.0256*** | -0.0294*** |
|  | (0.00988) | (0.00932) | (0.00986) |
| Urban | 0.0981*** | 0.0997*** | 0.0918*** |
|  | (0.0151) | (0.0146) | (0.0152) |
| Nearest Market (km) | 4.28e-06 | 7.68e-06 | -0.000129 |
|  | (0.000200) | (0.000192) | (0.000196) |
| Central Region | -0.0817*** | -0.0901*** | -0.0867*** |
|  | (0.0244) | (0.0241) | (0.0246) |
| South Region | -0.317*** | -0.315*** | -0.323*** |
|  | (0.0219) | (0.0217) | (0.0220) |
| SW Highlands Region | -0.255*** | -0.245*** | -0.263*** |
|  | (0.0254) | (0.0243) | (0.0254) |
| Lake Region | -0.0618*** | -0.0407* | -0.0650*** |
|  | (0.0220) | (0.0218) | (0.0220) |
| West Region | -0.178*** | -0.166*** | -0.178*** |
|  | (0.0242) | (0.0237) | (0.0241) |
| North Region | -0.0494* | -0.0724*** | -0.0556** |
|  | (0.0267) | (0.0260) | (0.0258) |
| South Highlands Region | -0.266*** | -0.261*** | -0.296*** |
|  | (0.0253) | (0.0251) | (0.0249) |
| Percent bushland | -0.0280 | -0.0304 | -0.0434 |
|  | (0.0274) | (0.0273) | (0.0267) |
| Percent cultivated land | -0.0151 | -0.00891 | -0.0304 |
|  | (0.0264) | (0.0263) | (0.0252) |
| Percent grassland | -0.0382 | -0.0400 | -0.0615** |
|  | (0.0289) | (0.0286) | (0.0281) |
| Percent woodland | -0.0810*** | -0.0600** | -0.102*** |
|  | (0.0294) | (0.0288) | (0.0286) |
| Percent natural forest | -0.0843 | -0.0550 | -0.108* |
|  | (0.0554) | (0.0548) | (0.0586) |
| District-level population density | 2.53e-05*** | 2.46e-05*** | 2.38e-05*** |
|  | (7.28e-06) | (7.22e-06) | (7.13e-06) |
| Percent economically active population | 0.148 | 0.224 | 0.182 |
|  | (0.233) | (0.230) | (0.234) |
| Percent voting population | -0.116 | -0.171 | -0.133 |
|  | (0.240) | (0.236) | (0.240) |
| Elevation | -3.22e-05** | -3.53e-05** | -2.92e-05** |
|  | (1.45e-05) | (1.41e-05) | (1.46e-05) |
| Slope | -0.00278 | -0.00536*** | -0.00193 |
|  | (0.00215) | (0.00205) | (0.00224) |
| Aridity Index | -2.19e-05*** | -2.50e-05*** | -2.29e-05*** |
|  | (2.86e-06) | (2.79e-06) | (2.89e-06) |
| 2007 | -0.0172 | -0.0176 | -0.0145 |
|  | (0.0127) | (0.0126) | (0.0127) |
| 2012 | -0.0527*** | -0.0536*** | -0.0503*** |
|  | (0.0114) | (0.0113) | (0.0113) |
| CBNRM dummy | -0.114*** | -0.0473** | -0.0276 |
|  | (0.0397) | (0.0232) | (0.0461) |
| CBNRM*2007 | 0.0459 | 0.0399 | 0.0555 |
|  | (0.0518) | (0.0322) | (0.0565) |
| CBNRM*2012 | 0.109** | 0.0659** | 0.0403 |
|  | (0.0495) | (0.0292) | (0.0545) |
| Constant | 1.007*** | 1.023*** | 1.031*** |
|  | (0.0620) | (0.0613) | (0.0617) |
|  |  |  |  |
| Observations | 16,723 | 18,237 | 16,586 |
| Pseudo R-squared | 0.029 | 0.03 | 0.03 |
| Robust standard errors in parentheses  *** p<0.01, ** p<0.05, * p<0.1 |  |  |  |
|  |  |  |  |
