# Supplementary material for: Impacts of Community-Based Natural Resource Management on Wealth, Food Security and Child Health in Tanzania
Source: PLoS One. 2015 Jul 17;10(7):e0133252. doi: 10.1371/journal.pone.0133252 (PMC4506085; doi:10.1371/journal.pone.0133252)
Supplement: S3 Table — This table shows full results of difference-in-differences models for JFM, CBFM and WMA, including all control variables. *** p<0.01, ** p<0.05, * p<0.1. (DOCX) [file pone.0133252.s004.docx]

**S4. Complete Difference-in-differences model for dependent variable: meat/fish/week**

| VARIABLES | JFM | CBFM | WMA |
| --- | --- | --- | --- |
| Wealth Index | 0.0673*** | 0.0687*** | 0.0670*** |
|  | (0.00224) | (0.00217) | (0.00222) |
| Number household members | 0.00314*** | 0.00309*** | 0.00305*** |
|  | (0.000720) | (0.000692) | (0.000720) |
| Number children under 5 | 0.0120*** | 0.0124*** | 0.0113*** |
|  | (0.00199) | (0.00189) | (0.00200) |
| Max number years education* | 0.00651*** | 0.00655*** | 0.00685*** |
|  | (0.000703) | (0.000664) | (0.000710) |
| Single adult head of hh | -0.00649 | -0.00392 | -0.00718 |
|  | (0.00549) | (0.00526) | (0.00554) |
| Female head of hh | 0.00559 | 0.00646 | 0.00357 |
|  | (0.00411) | (0.00394) | (0.00416) |
| Regional Avg 1999 Wealth | -0.0718*** | -0.0669*** | -0.0557*** |
|  | (0.0110) | (0.0101) | (0.0105) |
| Within 5km Protected Area | -0.00598 | -0.00453 | -0.00432 |
|  | (0.00388) | (0.00368) | (0.00398) |
| Within 5km Forest Reserve | 0.0201*** | 0.0199*** | 0.0179*** |
|  | (0.00373) | (0.00355) | (0.00373) |
| Urban | 0.0147*** | 0.0191*** | 0.0162*** |
|  | (0.00530) | (0.00516) | (0.00535) |
| Nearest Market (km) | 8.24e-05 | 0.000130* | 0.000195** |
|  | (8.29e-05) | (7.79e-05) | (8.14e-05) |
| Central Region | -0.0264*** | -0.0276*** | -0.0281*** |
|  | (0.00949) | (0.00952) | (0.00985) |
| South Region | -0.0239*** | -0.0204** | -0.0187** |
|  | (0.00825) | (0.00822) | (0.00829) |
| SW Highlands Region | -0.114*** | -0.109*** | -0.108*** |
|  | (0.0109) | (0.0103) | (0.0111) |
| Lake Region | -0.0466*** | -0.0386*** | -0.0393*** |
|  | (0.00853) | (0.00851) | (0.00862) |
| West Region | -0.0684*** | -0.0722*** | -0.0638*** |
|  | (0.00924) | (0.00917) | (0.00935) |
| North Region | 0.0985*** | 0.104*** | 0.0820*** |
|  | (0.0112) | (0.0108) | (0.0107) |
| South Highlands Region | -0.0160* | -0.00939 | 0.00305 |
|  | (0.00917) | (0.00920) | (0.00906) |
| Percent bushland | 0.0263*** | 0.0317*** | 0.0287*** |
|  | (0.00924) | (0.00919) | (0.00906) |
| Percent cultivated land | 0.0322*** | 0.0324*** | 0.0358*** |
|  | (0.00875) | (0.00868) | (0.00816) |
| Percent grassland | 0.0161 | 0.0205** | 0.0237** |
|  | (0.00981) | (0.00969) | (0.00951) |
| Percent woodland | 0.0174* | 0.0152 | 0.0182* |
|  | (0.0105) | (0.0102) | (0.0102) |
| Percent natural forest | 0.0679*** | 0.0569*** | 0.0874*** |
|  | (0.0186) | (0.0181) | (0.0194) |
| District-level population density | -2.61e-06 | -5.29e-06** | -5.94e-06** |
|  | (2.40e-06) | (2.36e-06) | (2.37e-06) |
| Percent economically active population | 0.0587 | -0.0113 | 0.0342 |
|  | (0.0579) | (0.0582) | (0.0590) |
| Percent voting population | -0.0811 | 0.000967 | -0.0772 |
|  | (0.0645) | (0.0642) | (0.0654) |
| Elevation | -1.66e-05*** | -1.92e-05*** | -2.50e-05*** |
|  | (5.41e-06) | (5.25e-06) | (5.51e-06) |
| Slope | -0.00575*** | -0.00509*** | -0.00621*** |
|  | (0.000816) | (0.000788) | (0.000881) |
| Aridity Index | 4.19e-06*** | 3.56e-06*** | 3.64e-06*** |
|  | (1.04e-06) | (1.02e-06) | (1.06e-06) |
| 2007 | 0.0241*** | 0.0232*** | 0.0216*** |
|  | (0.00526) | (0.00518) | (0.00527) |
| 2012 | 0.0379*** | 0.0370*** | 0.0358*** |
|  | (0.00446) | (0.00444) | (0.00445) |
| CBNRM dummy | 0.0573*** | 0.0149* | -0.00637 |
|  | (0.0120) | (0.00848) | (0.0160) |
| CBNRM*2007 | -0.0417** | -0.0181 | -0.0228 |
|  | (0.0168) | (0.0119) | (0.0197) |
| CBNRM*2012 | -0.0358** | -0.00310 | 0.0108 |
|  | (0.0153) | (0.0107) | (0.0187) |
| Constant | 0.822*** | 0.819*** | 0.844*** |
|  | (0.0232) | (0.0229) | (0.0230) |
|  |  |  |  |
| Observations | 16,748 | 18,263 | 16,609 |
| Pseudo R-squared | 0.008 | 0.008 | 0.008 |
| Robust standard errors in parentheses  *** p<0.01, ** p<0.05, * p<0.1 |  |  |  |
|  |  |  |  |
