# Supplementary material for: Impacts of Community-Based Natural Resource Management on Wealth, Food Security and Child Health in Tanzania
Source: PLoS One. 2015 Jul 17;10(7):e0133252. doi: 10.1371/journal.pone.0133252 (PMC4506085; doi:10.1371/journal.pone.0133252)
Supplement: S4 Table — This table shows full results of difference-in-differences models for JFM, CBFM and WMA, including all control variables. *** p<0.01, ** p<0.05, * p<0.1. (DOCX) [file pone.0133252.s005.docx]

**S5. Complete Difference-in-differences model for dependent variable: problems satisfying food needs**

| VARIABLES | JFM | CBFM | WMA |
| --- | --- | --- | --- |
| Wealth Index | 0.469*** | 0.476*** | 0.469*** |
|  | (0.0136) | (0.0132) | (0.0135) |
| Number household members | -0.00671* | -0.00877** | -0.00771* |
|  | (0.00400) | (0.00383) | (0.00401) |
| Number children under 5 | 0.0357*** | 0.0363*** | 0.0369*** |
|  | (0.0104) | (0.00993) | (0.0105) |
| Max number years education* | 0.0416*** | 0.0412*** | 0.0412*** |
|  | (0.00379) | (0.00359) | (0.00381) |
| Single adult head of hh | 0.0960*** | 0.0749** | 0.0983*** |
|  | (0.0303) | (0.0292) | (0.0304) |
| Female head of hh | -0.222*** | -0.214*** | -0.212*** |
|  | (0.0230) | (0.0221) | (0.0232) |
| Regional Avg 1999 Wealth | 0.102** | 0.0463 | 0.0974** |
|  | (0.0500) | (0.0471) | (0.0471) |
| Within 5km Protected Area | -0.0860*** | -0.0885*** | -0.0897*** |
|  | (0.0203) | (0.0193) | (0.0207) |
| Within 5km Forest Reserve | 0.0151 | 0.00949 | 0.0205 |
|  | (0.0198) | (0.0187) | (0.0197) |
| Urban | -0.0846*** | -0.0960*** | -0.0899*** |
|  | (0.0300) | (0.0295) | (0.0306) |
| Nearest Market (km) | -0.000829* | -0.000488 | -0.000947** |
|  | (0.000455) | (0.000420) | (0.000447) |
| Central Region | -0.0573 | -0.0469 | -0.0760 |
|  | (0.0506) | (0.0506) | (0.0516) |
| South Region | -0.0522 | -0.0597 | -0.0480 |
|  | (0.0472) | (0.0471) | (0.0474) |
| SW Highlands Region | 0.145*** | 0.160*** | 0.134*** |
|  | (0.0514) | (0.0498) | (0.0518) |
| Lake Region | 0.000196 | -0.0207 | -0.00933 |
|  | (0.0473) | (0.0472) | (0.0477) |
| West Region | -0.0729 | -0.104** | -0.0798 |
|  | (0.0503) | (0.0499) | (0.0505) |
| North Region | 0.210*** | 0.222*** | 0.223*** |
|  | (0.0553) | (0.0542) | (0.0531) |
| South Highlands Region | 0.0321 | 0.0208 | 0.0292 |
|  | (0.0502) | (0.0500) | (0.0501) |
| Percent bushland | -0.223*** | -0.219*** | -0.204*** |
|  | (0.0582) | (0.0577) | (0.0565) |
| Percent cultivated land | -0.271*** | -0.269*** | -0.263*** |
|  | (0.0553) | (0.0549) | (0.0520) |
| Percent grassland | -0.187*** | -0.189*** | -0.176*** |
|  | (0.0603) | (0.0596) | (0.0582) |
| Percent woodland | -0.184*** | -0.224*** | -0.164*** |
|  | (0.0621) | (0.0605) | (0.0599) |
| Percent natural forest | -0.198* | -0.211** | -0.210* |
|  | (0.105) | (0.104) | (0.114) |
| District-level population density | -3.61e-05** | -3.27e-05** | -3.05e-05** |
|  | (1.52e-05) | (1.51e-05) | (1.47e-05) |
| Percent economically active population | -1.617*** | -1.507*** | -1.508*** |
|  | (0.466) | (0.458) | (0.465) |
| Percent voting population | 1.453*** | 1.292*** | 1.297*** |
|  | (0.489) | (0.480) | (0.488) |
| Elevation | 2.22e-05 | 6.63e-06 | 3.44e-05 |
|  | (2.81e-05) | (2.74e-05) | (2.84e-05) |
| Slope | 0.0131*** | 0.0157*** | 0.00999** |
|  | (0.00424) | (0.00410) | (0.00449) |
| Aridity Index | 5.53e-06 | 9.44e-06* | 6.80e-06 |
|  | (5.24e-06) | (5.16e-06) | (5.33e-06) |
| 2007 | 0.0442* | 0.0366 | 0.0464* |
|  | (0.0258) | (0.0255) | (0.0257) |
| 2012 | 0.718*** | 0.713*** | 0.718*** |
|  | (0.0234) | (0.0232) | (0.0234) |
| CBNRM dummy | -0.0701 | -0.0539 | 0.185** |
|  | (0.0679) | (0.0463) | (0.0898) |
| CBNRM*2007 | 0.211** | 0.0205 | -0.114 |
|  | (0.0957) | (0.0645) | (0.111) |
| CBNRM*2012 | -0.101 | -0.141** | -0.565*** |
|  | (0.0849) | (0.0584) | (0.106) |
| Constant | 2.256*** | 2.238*** | 2.250*** |
|  | (0.137) | (0.136) | (0.136) |
|  |  |  |  |
| Observations | 16,723 | 18,236 | 16,585 |
| Pseudo R-squared | 0.083 | 0.083 | 0.085 |
| Robust standard errors in parentheses  *** p<0.01, ** p<0.05, * p<0.1 |  |  |  |
|  |  |  |  |
