# Supplementary material for: Impacts of Community-Based Natural Resource Management on Wealth, Food Security and Child Health in Tanzania
Source: PLoS One. 2015 Jul 17;10(7):e0133252. doi: 10.1371/journal.pone.0133252 (PMC4506085; doi:10.1371/journal.pone.0133252)
Supplement: S5 Table — This table shows full results of difference-in-differences models for JFM, CBFM and WMA, including all control variables. *** p<0.01, ** p<0.05, * p<0.1. (DOCX) [file pone.0133252.s006.docx]

**S6. Complete Difference-in-differences model for dependent variable: weight/age Z-score**

| VARIABLES | JFM | CBFM | WMA |
| --- | --- | --- | --- |
| Number household members | 0.00794 | 0.00892* | 0.00696 |
|  | (0.00525) | (0.00513) | (0.00524) |
| Number children under 5 | -0.0193 | -0.0237 | -0.0136 |
|  | (0.0153) | (0.0149) | (0.0153) |
| Child's age (months) | -0.0116*** | -0.0121*** | -0.0114*** |
|  | (0.000791) | (0.000759) | (0.000789) |
| Max number years education* | 0.0273* | 0.0296** | 0.0282** |
|  | (0.0141) | (0.0137) | (0.0143) |
| Femals | 0.0582** | 0.0540** | 0.0526* |
|  | (0.0270) | (0.0258) | (0.0270) |
| Single adult head of hh | -0.0502 | -0.0593 | -0.0369 |
|  | (0.0683) | (0.0661) | (0.0699) |
| Female head of hh | -0.00341 | -0.00488 | -0.0117 |
|  | (0.0400) | (0.0384) | (0.0405) |
| Shared Toilet | -0.0347 | -0.0299 | -0.0339 |
|  | (0.0358) | (0.0340) | (0.0362) |
| Tap Water | 0.109*** | 0.0932*** | 0.0830** |
|  | (0.0378) | (0.0352) | (0.0376) |
| Wealth Index | 0.212*** | 0.226*** | 0.203*** |
|  | (0.0254) | (0.0244) | (0.0250) |
| Regional Avg 1999 Wealth | 0.126* | 0.0614 | 0.158** |
|  | (0.0739) | (0.0711) | (0.0712) |
| Within 5km Protected Area | 0.0572 | 0.0584* | 0.0464 |
|  | (0.0353) | (0.0337) | (0.0356) |
| Within 5km Forest Reserve | 0.0371 | 0.0171 | 0.0368 |
|  | (0.0304) | (0.0284) | (0.0300) |
| Urban | -0.0524 | -0.0815 | -0.0352 |
|  | (0.0562) | (0.0523) | (0.0557) |
| Central Region | -0.131* | -0.0896 | -0.152* |
|  | (0.0790) | (0.0791) | (0.0837) |
| South Region | -0.241*** | -0.192*** | -0.186** |
|  | (0.0751) | (0.0731) | (0.0745) |
| SW Highlands Region | 0.0422 | 0.124 | 0.0420 |
|  | (0.0808) | (0.0771) | (0.0837) |
| Lake Region | 0.191*** | 0.233*** | 0.177** |
|  | (0.0718) | (0.0693) | (0.0744) |
| West Region | 0.152** | 0.152** | 0.135* |
|  | (0.0762) | (0.0738) | (0.0795) |
| North Region | -0.222** | -0.123 | -0.184** |
|  | (0.0898) | (0.0868) | (0.0901) |
| South Highlands Region | -0.0274 | 0.0142 | -0.0146 |
|  | (0.0786) | (0.0763) | (0.0814) |
| Percent bushland | 0.136 | 0.105 | 0.141 |
|  | (0.109) | (0.108) | (0.106) |
| Percent cultivated land | 0.0327 | -0.0123 | 0.0180 |
|  | (0.106) | (0.105) | (0.102) |
| Percent grassland | 0.0740 | 0.0612 | 0.0183 |
|  | (0.116) | (0.114) | (0.112) |
| Percent woodland | 0.0414 | 0.0598 | 0.0426 |
|  | (0.113) | (0.110) | (0.109) |
| Percent natural forest | -0.388* | -0.350* | -0.0251 |
|  | (0.210) | (0.195) | (0.215) |
| District-level population density | 3.93e-06 | 1.20e-05 | 1.64e-05 |
|  | (3.04e-05) | (3.00e-05) | (2.86e-05) |
| Percent economically active population | 2.831* | 2.802** | 2.494* |
|  | (1.462) | (1.391) | (1.436) |
| Percent voting population | -1.660 | -1.787 | -1.691 |
|  | (1.529) | (1.452) | (1.498) |
| Elevation | -4.17e-06 | -3.20e-05 | 2.95e-05 |
|  | (4.91e-05) | (4.80e-05) | (5.16e-05) |
| Slope | -0.0199*** | -0.0237*** | -0.0277*** |
|  | (0.00728) | (0.00672) | (0.00731) |
| Aridity Index | -4.35e-06 | -2.72e-06 | -6.01e-06 |
|  | (9.25e-06) | (8.80e-06) | (9.26e-06) |
| 2010 | 0.345*** | 0.339*** | 0.339*** |
|  | (0.0382) | (0.0377) | (0.0380) |
| CBNRM dummy | -0.346** | -0.189* | -0.0959 |
|  | (0.136) | (0.0963) | (0.149) |
| CBNRM*2010 | 0.254* | 0.151 | 0.244 |
|  | (0.152) | (0.105) | (0.167) |
| Constant | -1.887*** | -1.799*** | -1.689*** |
|  | (0.285) | (0.273) | (0.284) |
|  |  |  |  |
| Observations | 6,432 | 6,969 | 6,434 |
| R-squared | 0.100 | 0.101 | 0.092 |
| Robust standard errors in parentheses |  |  |  |
| *** p<0.01, ** p<0.05, * p<0.1 |  |  |  |
