# Supplementary material for: Impacts of Community-Based Natural Resource Management on Wealth, Food Security and Child Health in Tanzania
Source: PLoS One. 2015 Jul 17;10(7):e0133252. doi: 10.1371/journal.pone.0133252 (PMC4506085; doi:10.1371/journal.pone.0133252)
Supplement: S6 Table — This table shows full results of difference-in-differences models for JFM, CBFM and WMA, including all control variables. *** p<0.01, ** p<0.05, * p<0.1. (DOCX) [file pone.0133252.s007.docx]

**S7. Complete Difference-in-differences model for dependent variable: height/age Z-score**

| VARIABLES | JFM | CBFM | WMA |
| --- | --- | --- | --- |
| Number household members | 0.00303 | 0.00577 | 0.00335 |
|  | (0.00652) | (0.00639) | (0.00647) |
| Number children under 5 | -0.00847 | -0.0197 | -0.00545 |
|  | (0.0191) | (0.0187) | (0.0190) |
| Child's age (months) | -0.0171*** | -0.0173*** | -0.0166*** |
|  | (0.000973) | (0.000927) | (0.000967) |
| Max number years education* | 0.0360** | 0.0376** | 0.0446** |
|  | (0.0180) | (0.0172) | (0.0181) |
| Femals | 0.156*** | 0.140*** | 0.142*** |
|  | (0.0335) | (0.0319) | (0.0334) |
| Single adult head of hh | -0.0448 | -0.0501 | -0.0553 |
|  | (0.0812) | (0.0782) | (0.0829) |
| Female head of hh | 0.0288 | 0.0292 | 0.0395 |
|  | (0.0485) | (0.0464) | (0.0489) |
| Shared Toilet | -0.107** | -0.0955** | -0.0989** |
|  | (0.0440) | (0.0418) | (0.0443) |
| Tap Water | 0.138*** | 0.0919** | 0.114** |
|  | (0.0473) | (0.0444) | (0.0465) |
| Wealth Index | 0.225*** | 0.245*** | 0.237*** |
|  | (0.0323) | (0.0313) | (0.0315) |
| Regional Avg 1999 Wealth | -0.0762 | -0.141 | -0.0221 |
|  | (0.0900) | (0.0866) | (0.0866) |
| Within 5km Protected Area | 0.0517 | 0.0584 | 0.0456 |
|  | (0.0449) | (0.0425) | (0.0451) |
| Within 5km Forest Reserve | 0.0139 | -0.00512 | 0.0106 |
|  | (0.0375) | (0.0350) | (0.0368) |
| Urban | 0.000649 | -0.0378 | -0.0273 |
|  | (0.0670) | (0.0634) | (0.0660) |
| Central Region | 0.0429 | 0.0103 | 0.0414 |
|  | (0.0982) | (0.0979) | (0.105) |
| South Region | -0.371*** | -0.362*** | -0.321*** |
|  | (0.0916) | (0.0885) | (0.0909) |
| SW Highlands Region | -0.132 | -0.113 | -0.0876 |
|  | (0.106) | (0.0991) | (0.110) |
| Lake Region | 0.177* | 0.122 | 0.216** |
|  | (0.0904) | (0.0867) | (0.0938) |
| West Region | 0.245** | 0.119 | 0.269*** |
|  | (0.0970) | (0.0932) | (0.102) |
| North Region | 0.194* | 0.230** | 0.220** |
|  | (0.110) | (0.106) | (0.111) |
| South Highlands Region | -0.158 | -0.179* | -0.0978 |
|  | (0.0994) | (0.0951) | (0.103) |
| Percent bushland | 0.108 | 0.0729 | 0.135 |
|  | (0.131) | (0.129) | (0.126) |
| Percent cultivated land | 0.133 | 0.106 | 0.147 |
|  | (0.126) | (0.125) | (0.120) |
| Percent grassland | -0.00354 | -0.0358 | -0.00394 |
|  | (0.139) | (0.137) | (0.134) |
| Percent woodland | 0.0666 | 0.0708 | 0.117 |
|  | (0.138) | (0.133) | (0.133) |
| Percent natural forest | -0.375 | -0.449* | -0.0218 |
|  | (0.263) | (0.246) | (0.265) |
| District-level population density | 1.28e-05 | 3.17e-05 | 1.77e-05 |
|  | (3.40e-05) | (3.34e-05) | (3.20e-05) |
| Percent economically active population | 3.498** | 3.534** | 2.818* |
|  | (1.706) | (1.640) | (1.678) |
| Percent voting population | -1.978 | -2.345 | -1.581 |
|  | (1.811) | (1.724) | (1.777) |
| Elevation | -0.000120** | -0.000125** | -0.000119* |
|  | (6.01e-05) | (5.85e-05) | (6.38e-05) |
| Slope | -0.0173** | -0.0268*** | -0.0232*** |
|  | (0.00848) | (0.00783) | (0.00854) |
| Aridity Index | -1.60e-05 | -1.81e-05* | -2.52e-05** |
|  | (1.13e-05) | (1.04e-05) | (1.10e-05) |
| 2010 | 0.0733* | 0.0670 | 0.0570 |
|  | (0.0434) | (0.0428) | (0.0431) |
| CBNRM dummy | -0.456*** | -0.220** | -0.0934 |
|  | (0.143) | (0.112) | (0.151) |
| CBNRM*2010 | 0.314* | 0.184 | 0.133 |
|  | (0.169) | (0.123) | (0.176) |
| Constant | -2.441*** | -2.169*** | -2.293*** |
|  | (0.354) | (0.336) | (0.350) |
|  |  |  |  |
| Observations | 6,432 | 6,969 | 6,434 |
| R-squared | 0.096 | 0.095 | 0.091 |
| Robust standard errors in parentheses |  |  |  |
| *** p<0.01, ** p<0.05, * p<0.1 |  |  |  |
