# Supplementary material for: Impacts of Community-Based Natural Resource Management on Wealth, Food Security and Child Health in Tanzania
Source: PLoS One. 2015 Jul 17;10(7):e0133252. doi: 10.1371/journal.pone.0133252 (PMC4506085; doi:10.1371/journal.pone.0133252)
Supplement: S7 Table — This table shows full results of difference-in-differences models for JFM, CBFM and WMA, including all control variables. *** p<0.01, ** p<0.05, * p<0.1. (DOCX) [file pone.0133252.s008.docx]

**S8. Complete Difference-in-differences model for dependent variable:weight/height Z-score**

| VARIABLES | JFM | CBFM | WMA |
| --- | --- | --- | --- |
| Number household members | 0.00706 | 0.00660 | 0.00569 |
|  | (0.00537) | (0.00525) | (0.00538) |
| Number children under 5 | -0.00898 | -0.00625 | -0.00345 |
|  | (0.0160) | (0.0155) | (0.0161) |
| Child's age (months) | -0.00229** | -0.00273*** | -0.00247*** |
|  | (0.000891) | (0.000854) | (0.000891) |
| Max number years education* | 0.0115 | 0.0127 | 0.00819 |
|  | (0.0157) | (0.0151) | (0.0161) |
| Femals | -0.0174 | -0.0121 | -0.0150 |
|  | (0.0289) | (0.0275) | (0.0289) |
| Single adult head of hh | -0.0293 | -0.0416 | -0.00429 |
|  | (0.0735) | (0.0706) | (0.0757) |
| Female head of hh | -0.0299 | -0.0303 | -0.0523 |
|  | (0.0432) | (0.0416) | (0.0429) |
| Shared Toilet | 0.0350 | 0.0345 | 0.0320 |
|  | (0.0378) | (0.0358) | (0.0380) |
| Tap Water | 0.0421 | 0.0609 | 0.0297 |
|  | (0.0402) | (0.0374) | (0.0400) |
| Wealth Index | 0.105*** | 0.109*** | 0.0768*** |
|  | (0.0272) | (0.0263) | (0.0267) |
| Regional Avg 1999 Wealth | 0.251*** | 0.216*** | 0.237*** |
|  | (0.0792) | (0.0757) | (0.0768) |
| Within 5km Protected Area | 0.0508 | 0.0407 | 0.0355 |
|  | (0.0387) | (0.0366) | (0.0389) |
| Within 5km Forest Reserve | 0.0394 | 0.0298 | 0.0430 |
|  | (0.0320) | (0.0300) | (0.0318) |
| Urban | -0.0698 | -0.0810 | -0.0203 |
|  | (0.0585) | (0.0559) | (0.0582) |
| Central Region | -0.202** | -0.126 | -0.230** |
|  | (0.0873) | (0.0865) | (0.0893) |
| South Region | -0.0485 | 0.0190 | -0.0107 |
|  | (0.0784) | (0.0761) | (0.0783) |
| SW Highlands Region | 0.189** | 0.286*** | 0.156* |
|  | (0.0891) | (0.0845) | (0.0912) |
| Lake Region | 0.136* | 0.236*** | 0.0819 |
|  | (0.0788) | (0.0761) | (0.0807) |
| West Region | 0.0159 | 0.119 | -0.0269 |
|  | (0.0836) | (0.0806) | (0.0857) |
| North Region | -0.457*** | -0.352*** | -0.415*** |
|  | (0.0961) | (0.0927) | (0.0968) |
| South Highlands Region | 0.105 | 0.190** | 0.0809 |
|  | (0.0879) | (0.0859) | (0.0893) |
| Percent bushland | 0.0787 | 0.0669 | 0.0656 |
|  | (0.116) | (0.114) | (0.112) |
| Percent cultivated land | -0.0976 | -0.137 | -0.127 |
|  | (0.113) | (0.111) | (0.108) |
| Percent grassland | 0.0906 | 0.0999 | 0.00836 |
|  | (0.125) | (0.123) | (0.121) |
| Percent woodland | -0.0230 | 0.000926 | -0.0661 |
|  | (0.121) | (0.117) | (0.117) |
| Percent natural forest | -0.235 | -0.110 | -0.0198 |
|  | (0.237) | (0.215) | (0.245) |
| District-level population density | -1.28e-05 | -1.56e-05 | 4.77e-06 |
|  | (2.96e-05) | (2.91e-05) | (2.83e-05) |
| Percent economically active population | 0.921 | 0.831 | 0.913 |
|  | (1.512) | (1.438) | (1.490) |
| Percent voting population | -0.553 | -0.459 | -0.827 |
|  | (1.593) | (1.507) | (1.562) |
| Elevation | 6.55e-05 | 3.28e-05 | 0.000117** |
|  | (5.32e-05) | (5.19e-05) | (5.49e-05) |
| Slope | -0.0134* | -0.0107 | -0.0192** |
|  | (0.00757) | (0.00697) | (0.00762) |
| Aridity Index | 7.77e-06 | 9.29e-06 | 1.20e-05 |
|  | (9.72e-06) | (9.23e-06) | (9.70e-06) |
| 2010 | 0.285*** | 0.284*** | 0.292*** |
|  | (0.0373) | (0.0367) | (0.0372) |
| CBNRM dummy | -0.136 | -0.0746 | -0.0375 |
|  | (0.144) | (0.0921) | (0.134) |
| CBNRM*2010 | 0.0873 | 0.0288 | 0.192 |
|  | (0.165) | (0.102) | (0.163) |
| Constant | -0.523* | -0.585** | -0.390 |
|  | (0.296) | (0.284) | (0.297) |
|  |  |  |  |
| Observations | 6,432 | 6,969 | 6,434 |
| R-squared | 0.043 | 0.045 | 0.039 |

Robust standard errors in parentheses

*** p<0.01, ** p<0.05, * p<0.1
