# Supplementary material for: Impacts of Community-Based Natural Resource Management on Wealth, Food Security and Child Health in Tanzania
Source: PLoS One. 2015 Jul 17;10(7):e0133252. doi: 10.1371/journal.pone.0133252 (PMC4506085; doi:10.1371/journal.pone.0133252)
Supplement: S8 Table — This table shows full results of difference-in-differences models for JFM, CBFM and WMA, including all control variables for those households that directly intersect the CBNRM village census tracts. *** p<0.01, ** p<0.05, * p<0.1. (DOCX) [file pone.0133252.s009.docx]

**S9.** **Differences-in-differences model results for wealth, food security and health outcomes using a sample of households that directly intersect the CBNRM village census tracts (ie no 5km buffer)**

|  | 0 KM distance | | | 5 KM distance | | |
| --- | --- | --- | --- | --- | --- | --- |
|  | JFM | CBFM | WMA | JFM | CBFM | WMA |
| Wealth index | 0.0131 | -0.214*** | -0.179** | 0.0675 | -0.0624** | -0.0278 |
|  | (0.0564) | (0.0507) | (0.0718) | (0.0481) | (0.0294) | (0.0702) |
| Meals/day | 0.166** | 0.151*** | -0.0922 | 0.109** | 0.0659** | 0.0403 |
|  | (0.0670) | (0.0485) | (0.0860) | (0.0495) | (0.0292) | (0.0545) |
| Meat/fish per week (# of times) | -0.101*** | -0.0240 | -0.00713 | -0.0358** | -0.00310 | 0.0108 |
|  | (0.0214) | (0.0177) | (0.0305) | (0.0153) | (0.0107) | (0.0187) |
| Problems satisfying food needs last year | -0.148 | -0.302*** | -0.582*** | -0.101 | -0.141** | -0.565*** |
|  | (0.118) | (0.0989) | (0.167) | (0.0849) | (0.0584) | (0.106) |
| Weight/age | 27.76 | 4.047 | 27.02 | 25.44* | 15.08 | 24.40 |
|  | (25.15) | (16.32) | (35.37) | (15.18) | (10.45) | (16.71) |
| Height/age | 22.03 | -2.673 | 38.61 | 31.44* | 18.36 | 13.29 |
|  | (24.02) | (18.58) | (42.16) | (16.89) | (12.32) | (17.57) |
| Weight/height | 20.53 | 8.815 | 5.242 | 8.729 | 2.883 | 19.15 |
|  | (26.16) | (15.81) | (28.92) | (16.48) | (10.23) | (16.31) |

The results from the original, full sample using a 5km buffer are included for comparison. The coefficient values are similar when implementing a 0km and a 5km buffer. The 0km results are stronger in magnitude and significance because this sample includes fewer households that are not CBNRM. When expanding the radius 5km around the DHS GPS points, more households that are not truly in CBNRM will be included as CBNRM, diluting the CBNRM “effect” and biasing results against finding a significant effect.
